# The Impact of a One-Dose versus Two-Dose Oral Cholera Vaccine Regimen in Outbreak Settings: A Modeling Study

**DOI:** 10.1371/journal.pmed.1001867

**Published:** 2015-08-25

**Authors:** Andrew S. Azman, Francisco J. Luquero, Iza Ciglenecki, Rebecca F. Grais, David A. Sack, Justin Lessler

**Affiliations:** 1 Department of Epidemiology, Johns Hopkins Bloomberg School of Public Health, Baltimore, Maryland, United States of America; 2 Epicentre, Paris, France; 3 Médecins Sans Frontières, Geneva, Switzerland; 4 Department of International Health, Johns Hopkins Bloomberg School of Public Health, Baltimore, Maryland, United States of America; The National Institute for Public Health and the Environment, NETHERLANDS

## Abstract

**Background:**

In 2013, a stockpile of oral cholera vaccine (OCV) was created for use in outbreak response, but vaccine availability remains severely limited. Innovative strategies are needed to maximize the health impact and minimize the logistical barriers to using available vaccine. Here we ask under what conditions the use of one dose rather than the internationally licensed two-dose protocol may do both.

**Methods and Findings:**

Using mathematical models we determined the minimum relative single-dose efficacy (MRSE) at which single-dose reactive campaigns are expected to be as or more effective than two-dose campaigns with the same amount of vaccine. Average one- and two-dose OCV effectiveness was estimated from published literature and compared to the MRSE. Results were applied to recent outbreaks in Haiti, Zimbabwe, and Guinea using stochastic simulations to illustrate the potential impact of one- and two-dose campaigns. At the start of an epidemic, a single dose must be 35%–56% as efficacious as two doses to avert the same number of cases with a fixed amount of vaccine (i.e., MRSE between 35% and 56%). This threshold decreases as vaccination is delayed. Short-term OCV effectiveness is estimated to be 77% (95% CI 57%–88%) for two doses and 44% (95% CI −27% to 76%) for one dose. This results in a one-dose relative efficacy estimate of 57% (interquartile range 13%–88%), which is above conservative MRSE estimates. Using our best estimates of one- and two-dose efficacy, we projected that a single-dose reactive campaign could have prevented 70,584 (95% prediction interval [PI] 55,943–86,205) cases in Zimbabwe, 78,317 (95% PI 57,435–100,150) in Port-au-Prince, Haiti, and 2,826 (95% PI 2,490–3,170) cases in Conakry, Guinea: 1.1 to 1.2 times as many as a two-dose campaign. While extensive sensitivity analyses were performed, our projections of cases averted in past epidemics are based on severely limited single-dose efficacy data and may not fully capture uncertainty due to imperfect surveillance data and uncertainty about the transmission dynamics of cholera in each setting.

**Conclusions:**

Reactive vaccination campaigns using a single dose of OCV may avert more cases and deaths than a standard two-dose campaign when vaccine supplies are limited, while at the same time reducing logistical complexity. These findings should motivate consideration of the trade-offs between one- and two-dose campaigns in resource-constrained settings, though further field efficacy data are needed and should be a priority in any one-dose campaign.

## Abstract 2

Modelling the efficacy of single-dose cholera vaccination in resource-limited settings.

## Editors' Summary

**Background:**

Cholera—a bacterial gut infection caused by *Vibrio cholerae*—is a major global killer. Every year, epidemics (outbreaks) of cholera make 2 to 3 million people ill and kill about 100,000 people. People get cholera by eating food or drinking water contaminated with feces from an infected person, so cholera epidemics occur in places with poor sanitation such as slums and refugee camps. Earthquakes, floods, and other natural disasters that disrupt water and sanitation systems can also trigger cholera epidemics. Most people who become infected with *V*. *cholerae* have no or mild symptoms, but they may shed bacteria in their feces for up to two weeks. Other infected individuals develop severe diarrhea, producing profuse watery feces. The standard treatment for cholera is replacement of the fluids and salts lost through diarrhea by drinking an oral rehydration fluid or, in the worst cases, by fluid replacement directly into a vein. With prompt treatment, less than 1% of patients die, but untreated patients with severe cholera can die from dehydration within hours of developing symptoms.

**Why Was This Study Done?:**

The best way to control cholera is to ensure that everyone has access to safe water and good sanitation, but this is often impossible in poor countries, in refugee camps, or after natural disasters. In 2013, the World Health Organization created a global stockpile of an oral cholera vaccine (a preparation given by mouth that stimulates the immune system to attack *V*. *cholerae*) for use in cholera outbreaks. The licensed protocol for the currently stockpiled vaccine requires two doses of the vaccine to be given two weeks apart, but it can be difficult to ensure that everyone at risk of infection receives two doses. Moreover, the stockpile contains only one to two million doses of vaccine, which would have been insufficient to protect every individual at risk of infection in several recent cholera outbreaks. Here, the researchers use mathematical modeling to investigate whether one dose of oral cholera vaccine, rather than two doses, could be used to maximize the health impact of cholera vaccination and minimize logistical barriers to cholera vaccination during cholera outbreaks.

**What Did the Researchers Do and Find?:**

The researchers used cholera transmission models to determine the “minimum relative single-dose efficacy” (MRSE), the threshold at which single-dose vaccination campaigns begun after an outbreak has started (reactive vaccination) are expected to be as or more effective than two-dose campaigns with the same amount of vaccine. The researchers report that, at the start of an epidemic, the MRSE is between 35% and 56%. That is, a single dose of vaccine must be at least 35%–56% as efficacious as two doses to avert the same number of cases with a fixed amount of vaccine. By searching the literature, the researchers estimated that the short-term protection against infection provided by oral cholera vaccines is 77% for two doses and 44% for one dose—a one-dose relative efficacy of 57%, which is above the MRSE estimate. Finally, the researchers used their models to project that, in three recent cholera outbreaks, a single-dose campaign could have prevented between 1.1 and 1.2 times more cases of cholera than a two-dose campaign using the same amount of vaccine.

**What Do These Findings Mean?:**

The finding that the relative single-dose efficacy of oral cholera vaccination is above the estimated MRSE suggests that one-dose reactive vaccination campaigns might avert more cases and deaths than a standard two-dose campaign when vaccine supplies are limited. The accuracy of this and other study findings is limited, however, by the assumptions used to build the mathematical models and by the quality of the data used to run them. In particular, a lack of data on the efficacy of single-dose vaccination limits the ability to apply these findings. Thus, before one-dose campaigns are used widely, more data on the effectiveness on one-dose vaccination must be obtained. Notably, by increasing herd immunity (the vaccination of a significant portion of a population provides some protection for individuals in the population who have not been vaccinated), one-dose campaigns are likely to provide better population-level protection than two-dose campaigns. On the other hand, the individual who is given one rather than two vaccine doses is more vulnerable to cholera illness if exposed to cholera-causing bacteria. Strategies that balance the trade-off between individual- and population-level benefits must be carefully considered to ensure the best future use of the oral cholera vaccine stockpile. Moreover, every effort should be made to increase the size and availability of this stockpile.

**Additional Information:**

This list of resources contains links that can be accessed when viewing the PDF on a device or via the online version of the article at http://dx.doi.org/10.1371/journal.pmed.1001867.

- The World Health Organization provides information about cholera in several languages, including technical information about cholera vaccination and the oral cholera vaccine stockpile
- The US Centers for Disease Control and Prevention also provides information about cholera for the public, medical professionals, and travelers
- The UK National Health Service Choices website provides information about cholera and cholera vaccination; it also provides information, including a simple animation, about how vaccines work and herd immunity
- MedlinePlus provides links to further resources about cholera
- The not-for-profit organization Médecins Sans Frontières is tackling several cholera outbreaks around the world; its website includes a description of the recent vaccination of refugees against cholera in Tanzania
- A personal story about the 2008–2009 cholera outbreak in Zimbabwe is available from UNICEF
- StopCholera.org provides detailed information on oral cholera vaccines useful for public health officials, scientists, and clinicians

## Introduction

Despite years of control efforts, cholera remains a major killer worldwide, causing an estimated 2 to 3 million cases and 100,000 deaths each year [1]. Recently, large outbreaks in Haiti, West Africa, and Zimbabwe have renewed the focus on cholera as a controllable threat to public health [2–4]. Control efforts have traditionally focused on improving case management and increasing the availability of safe drinking water. A new breed of oral cholera vaccines (OCVs) may be an important addition to the cholera control toolbox, particularly in outbreaks, where rapid action is required [5].

In 2010, the World Health Organization recommended consideration of OCV use in outbreak response [6]. Use of vaccine prior to an outbreak is considered “preemptive,” while vaccination after an outbreak begins is referred to as “reactive.” In 2013, the World Health Organization created a global vaccine stockpile, managed by the International Coordinating Group, to support rapid deployment of OCV in reactive campaigns. Despite the establishment of this stockpile, the number of OCV doses available (1 to 2 million at the time of writing) is dwarfed by the estimated 1.5 billion at risk for cholera globally and would not have covered the at-risk population in many recent outbreaks (e.g., 3.2 million doses would be needed to give a full course of vaccine to the entire population of Harare, Zimbabwe) [1,7]. OCV supplies are likely to remain limited for many years, and creative approaches are needed to maximize the public health benefit of this limited resource.

The internationally licensed protocol for Shanchol (Shantha Biotechnics), the only vaccine currently provided by the stockpile, is two doses given 2 wk apart. In the face of limited supply, a modified one-dose schedule may have an equal or greater impact on an epidemic while decreasing the logistical complexity and costs of reactive OCV campaigns. There are logistical costs involved in tracking OCV recipients and appropriately targeting the second dose. At the same time, during the interval between the first and second dose, the epidemic progresses, reducing the number of people who can be protected by vaccination. Hence, while reducing logistical challenges, a one-dose protocol benefits from quickly providing at least partial cholera protection to as many people as possible.

Reduced-dose protocols have been considered in the past to maximize the impact of limited supplies of meningococcal polysaccharide vaccine and inactivated poliovirus vaccine [8,9]. Consideration of a less efficacious regimen—as is likely to be the case with one dose compared to two doses of OCV—may involve a trade-off between individual- and population-level benefits. What is best for the individual may not always be best for the population. Exploring the conditions where these two objectives may be at odds with one another can provide public health decision-makers with data to make better-informed vaccine allocation decisions.

The relative impact on morbidity and mortality of a single-dose reactive OCV campaign, compared to a two-dose campaign with the same number of total doses, will depend on relative vaccine efficacy, the magnitude of indirect vaccine (“herd”) protection, and the local epidemiology of cholera outbreaks. Here we explore the relative efficacy needed by a single-dose campaign to prevent at least as many cholera cases as a two-dose campaign, and explore the impact OCV use could have had in recent cholera outbreaks in Zimbabwe, Haiti, and Guinea. Our primary aim is to determine the threshold for the relative efficacy of one dose of OCV where one-dose reactive campaigns are expected to have at least as large an impact as two-dose campaigns with the same amount of vaccine. If single-dose reactive campaigns are as effective in averting cases as two-dose campaigns, single-dose campaigns could be a boon to public health by efficiently using limited resources while reducing the logistical challenges of reactive vaccination.

## Methods

### Meta-Analysis of Vaccine Effectiveness

We estimated the average short-term (less than 1 y) effectiveness of one and two doses of OCV using published data from both randomized and observational (intervention) studies. We included estimates for both internationally licensed vaccines, Shanchol and Dukoral, because of the similar composition in the main antigens and the similar efficacy estimates from the literature, and to make the results of this analysis more generalizable. We estimated the average short-term one- and two-dose efficacy using a random intercept linear regression model [10]. Confidence intervals for the ratio of these two efficacy estimates were constructed following Newcombe [11].

### Cholera Transmission Models

**Fig 1:**
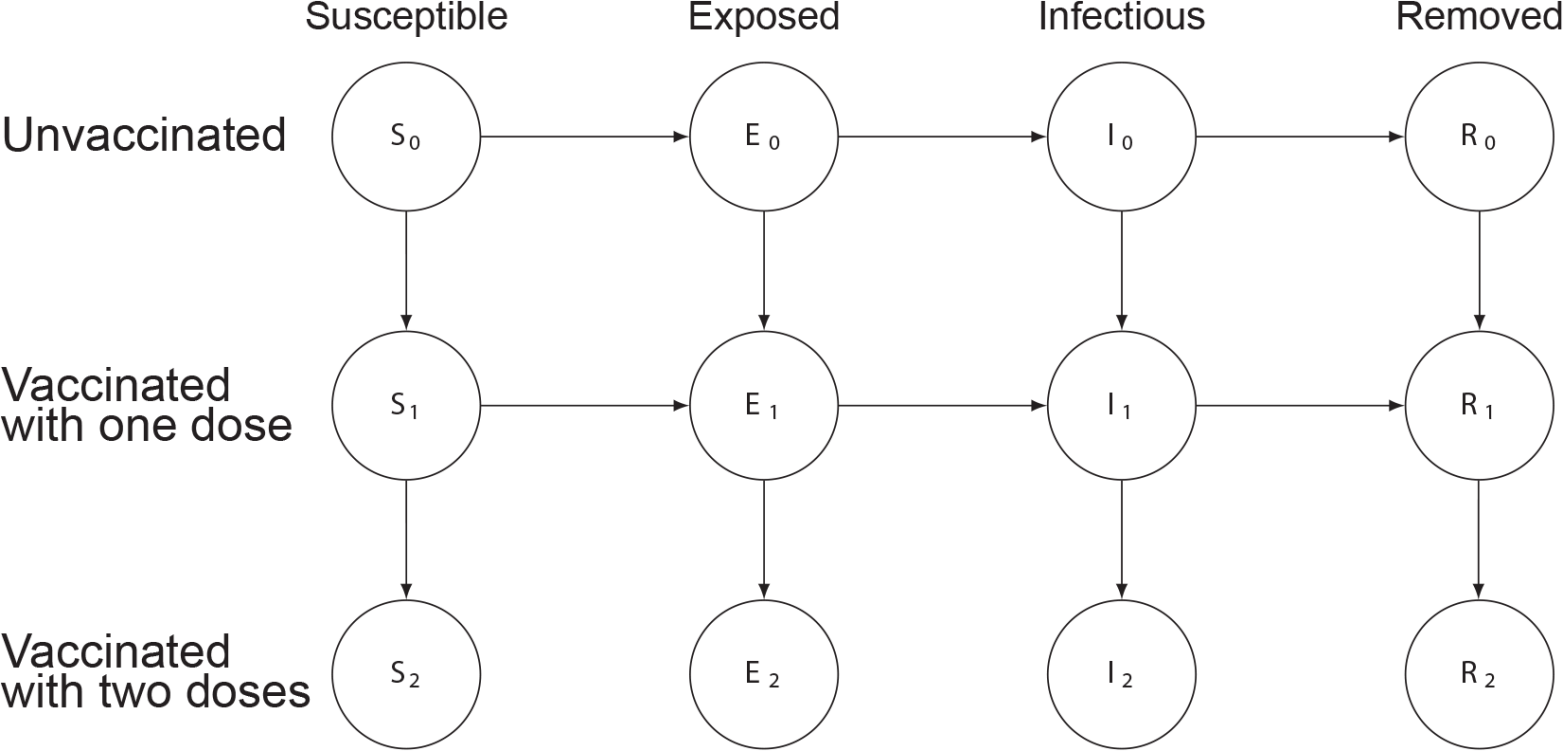
Illustration of the susceptibility-reducing vaccine model. Circles represent states, with the variable letters representing susceptible, exposed, infectious, and removed states and the subscripts indicating the number of doses of vaccine those in that state have received. Arrows between states represent rates of transition between states, with values indicated in Table 1. More details can be found in S1 Text.

**Fig 2:**
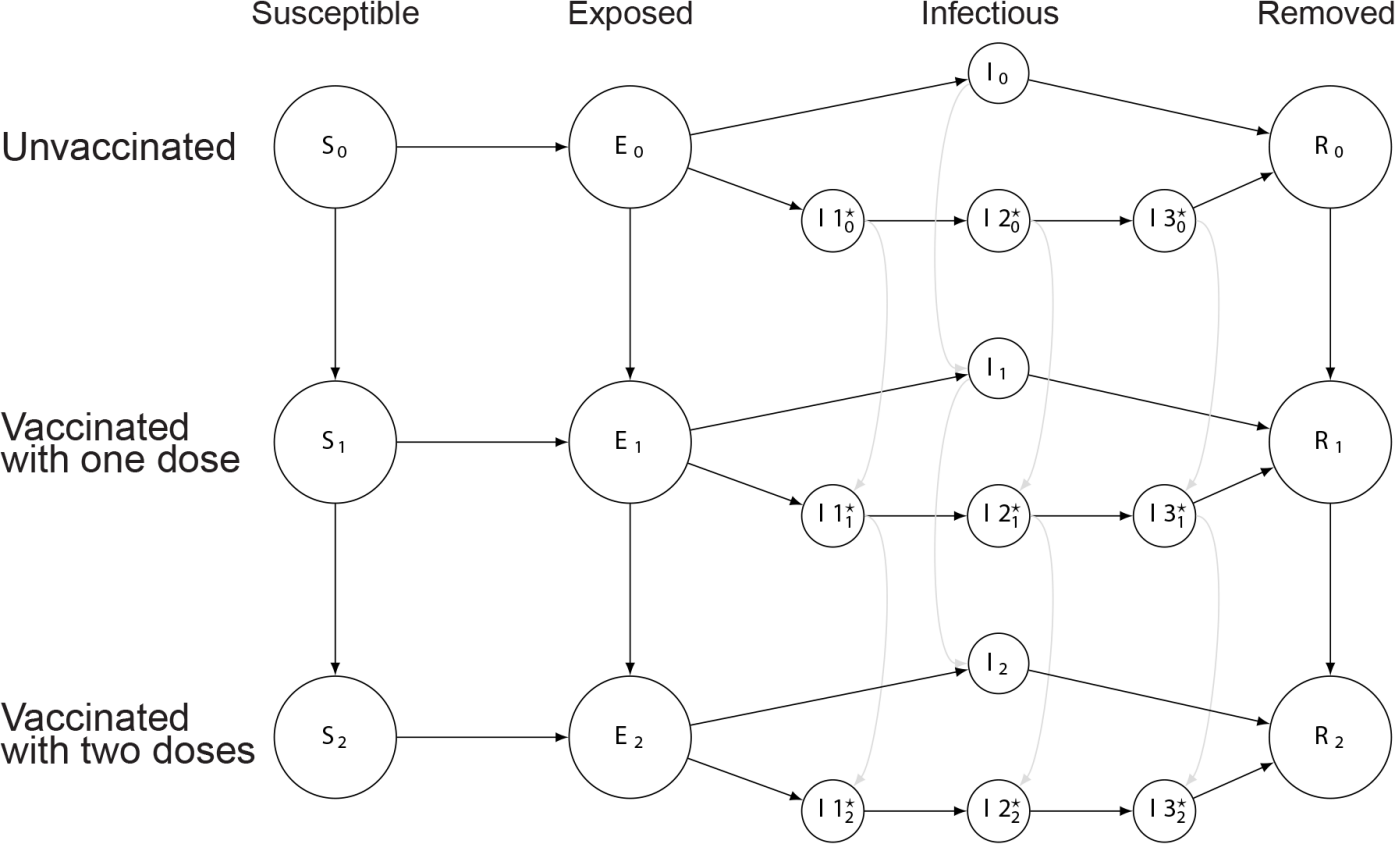
Illustration of model including fast and slow cholera transmission. Circles represent states, with the variable letters representing susceptible, exposed, infectious, and removed states and the subscripts indicating the number of doses of vaccine those in that state have received. In the infectious states, those with a star indicate states involved with the slow (environmentally mediated) transmission pathway. Arrows between states represent rates of transition between states. More details can be found in S1 Text.

We constructed deterministic compartmental transmission models where individuals are susceptible, exposed but not infectious, infectious, or removed from the system due to immunity or death (an SEIR model) [12]. Although some evidence suggests that cholera vaccine reduces susceptibility to disease and illness severity [13], there is much uncertainty about how the vaccine protects individuals. To capture this uncertainty in our results, we modeled vaccines providing protection by completely preventing infection in a percent of vaccinees (an “all-or-nothing” vaccine), reducing susceptibility among all vaccinees by a percent (a susceptibility-reducing vaccine; Fig 1), or preventing severe disease in a percent of vaccinees (a severity-reducing vaccine) [14]. In the last case, mild and asymptomatic cases are presumed to be less infectious. In addition, we considered a model that included both “fast” (i.e., person-to-person) and “slow” (i.e., environmentally mediated) transmission pathways to understand how mixtures of modes of transmission may impact our findings (Fig 2) [15]. Epidemic simulations were seeded with a single case unless otherwise noted. See Table 1 and S3 Text for model structure and parameter details.

**Table 1:** Core parameters used in deterministic transmission models.

Parameter	Description	Value	Source
1/σ	Mean latent period	1.41 d	[16]
1/γ	Mean duration of infectiousness	2.0 d	[17]
ρ_1_	Vaccination rate for dose 1	Varied^§^	[18]
ρ_2_	Vaccination rate for dose 2	Varied^§^	[18]
β	Transmission parameter	0.654 d^−1^	Calibrated
θ_1_	One-dose vaccine efficacy	Varied	Meta-analysis, S1 Fig
θ_2_	Two-dose vaccine efficacy	Varied	Meta-analysis, S1 Fig

Models described in more detail in S1 Text.

^§^Vaccination rates varied to keep the duration of the campaigns constant.

### Vaccination Scenarios

In a hypothetical cholera epidemic (based on the 2008 epidemic in Bissau, Guinea-Bissau [19]), we considered scenarios where reactive vaccination (1) did not take place, (2) used a one-dose protocol, or (3) used a two-dose protocol. The distribution of all OCV doses was assumed to take 10 d, in either a single 10-d round (for one-dose campaigns) or two 5-d rounds occurring 2 wk apart (for two-dose campaigns) (S1 Fig). Protection from each dose was assumed to begin immediately after vaccination, though this assumption was relaxed in sensitivity analyses (S4 Text). Individuals who received two doses had the same protection as those receiving a single dose in the time between their first and second dose. We simulated start dates for vaccination ranging from the onset date of the first case to the final day of the outbreak. We considered a single dose that was from 0% to 100% as efficacious as two doses, and the use of 1,000 to 500,000 doses of vaccine in a population of 500,000 individuals.

### Minimum Relative Single-Dose Efficacy

Our main objective was to determine how much less effective a single dose of OCV can be and still have an equivalent public health impact as a two-dose regimen provided to half as many people. Since the efficacy of any regimen likely varies across epidemiologic settings, we chose to formulate our results in a relative manner (i.e., our main results are not dependent on a specific value for two-dose efficacy) and to define the relative single-dose efficacy (RSE) as

$$\text{RSE}=\frac{\text{one-dose efficacy}}{\text{two-dose efficacy}}\times 100$$ (1)

The primary outcome considered is the minimum RSE (MRSE). The MRSE is defined as the RSE above which a single-dose campaign prevents as many or more cases of cholera than a two-dose campaign using the same number of doses. For example, suppose that distributing 100,000 doses of OCV as a single dose would elicit immunity to cholera in 40,000 (40% efficacy) and prevent 1,000 cholera cases, while distributing the same 100,000 doses as a two-dose protocol to 50,000 would also elicit immunity in 40,000 individuals (80% efficacy) and prevent 1,000 cholera cases. Then the MRSE is 40/80 × 100 = 50%. Thus, in this hypothetical setting, any one-dose campaign where a single dose of the vaccine is at least 50% as efficacious as a two-dose regimen would prevent as many or more cases than a two-dose campaign with the same total amount of vaccine. The lower the value of the MRSE, the less protective a single-dose vaccine needs to be to still avert at least as many cases as a two-dose campaign covering half as many people.

**Fig 3:**
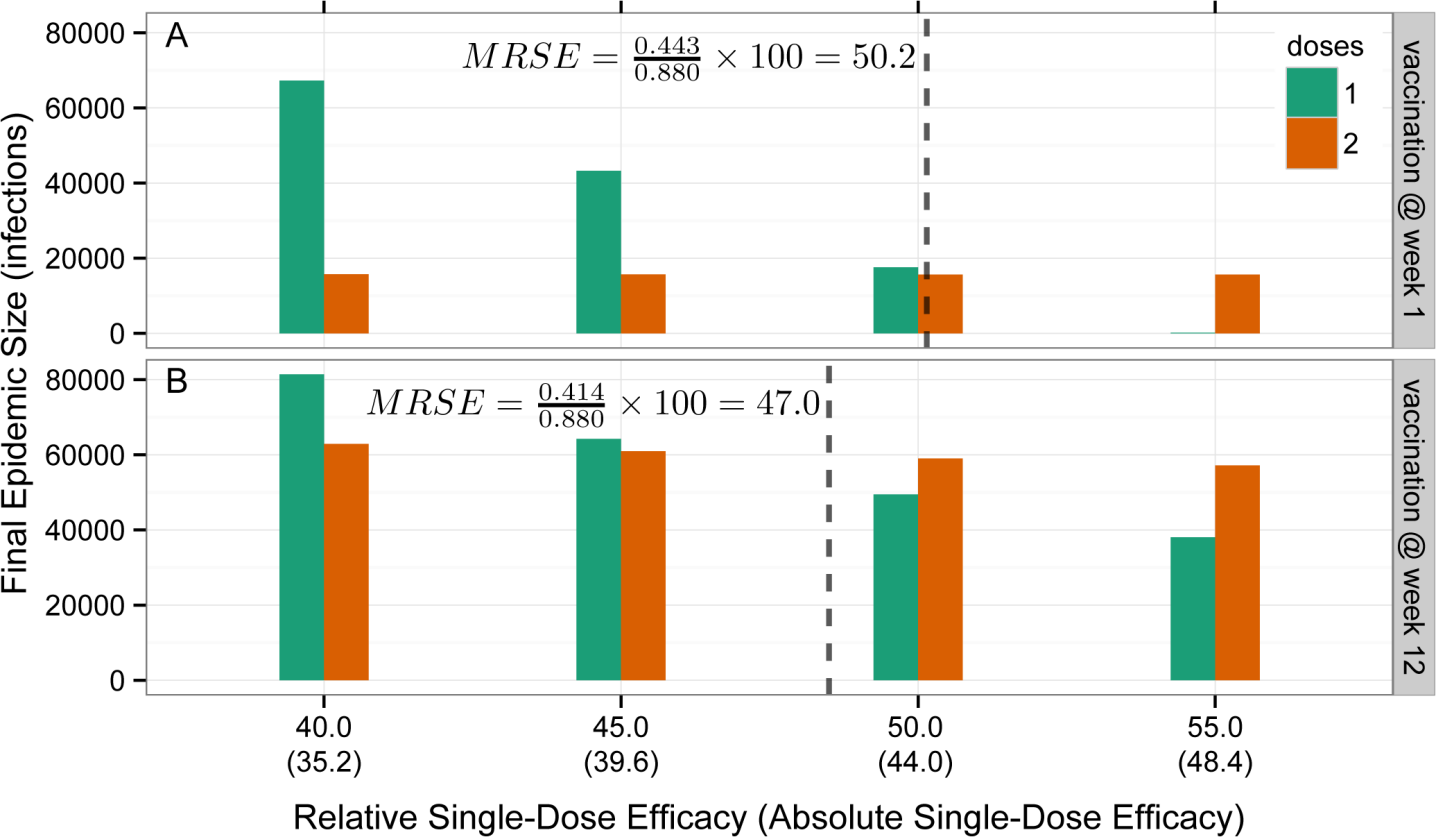
Illustration of minimum relative single-dose efficacy in example vaccination campaigns starting at week 1 and week 12. Bars represent the final epidemic size for one-dose (green) and two-dose (orange) campaigns as a function of the RSE or the absolute single-dose efficacy (assuming a two-dose efficacy of 88%, in parentheses) on the *x*-axis for campaigns starting at week 1 (A) and week 12 (B) of the outbreak. The MRSE (dashed vertical line) is the RSE at which the final epidemic sizes for one- and two-dose campaigns are equal.

We estimated the MRSE for each model and vaccine coverage by simulating one- and two-dose campaigns with relative one-dose efficacies ranging from 0% to 100% and determining the minimum one-dose efficacy resulting in equivalent epidemic sizes from both vaccination protocols. See Fig 3 and S2 Text for additional details on MRSE.

### Potential Benefits of a Single-Dose Oral Cholera Vaccine Campaign in Recent Epidemics

To explore the impact vaccination might have had in recent notable epidemics, we fit stochastic versions of our model to the 2008–2009 epidemic in Zimbabwe (we used country-wide data because of the lack of high-quality data from Harare), the first two waves of the cholera epidemic in Port-au-Prince, Haiti (October 2010 through September 2011), and the 2012 cholera epidemic in Conakry, Guinea [18,20,21]. We estimated the parameters (see S3 Text) for each model (separately for each epidemic) within a likelihood framework using maximum likelihood via iterated filtering (MIF), a method designed for inference on partially observed nonlinear stochastic dynamic systems [22]. Using these fitted models, we simulated the effects of hypothetical one- and two-dose reactive OCV campaigns with vaccine efficacy based upon our meta-analysis. In each case we selected a plausible vaccination start date based upon (unpublished) operational reports from Médecins Sans Frontières–supported Ministry of Health campaigns and experience from previous campaigns in Guinea and South Sudan. Through 100,000 stochastic runs, these simulations capture uncertainty in the course of the epidemics and cholera surveillance, although they do not capture uncertainty in model structure or vaccine mechanism. Additional details are provided in S3 Text.

All analyses were done in the R statistical package [23]. Full details on the methods and data used in all analyses are available in S1–S4 Texts.

## Results

### Current Estimates of Oral Cholera Vaccine Effectiveness

Through a literature search we identified six estimates of the efficacy or effectiveness of the killed OCV [5,24–28] against culture-confirmed cholera (except [5], which used rapid tests for confirmation) where protection was assessed 3–12 mo from the time of vaccination. These studies included both large placebo-controlled randomized clinical trials with 16 to 88 cases (across both placebo and intervention arms) to field effectiveness studies with 40 to 43 cases. We excluded the estimate from Matlab, Bangladesh [27], where the study administered three doses of a killed whole cell vaccine with a recombinant B subunit (similar to Dukoral), since the protocol was inconsistent with those of the other studies. Because of limited data, we combined estimates of effectiveness (from field trials) and efficacy (from randomized trials).

**Fig 4:**
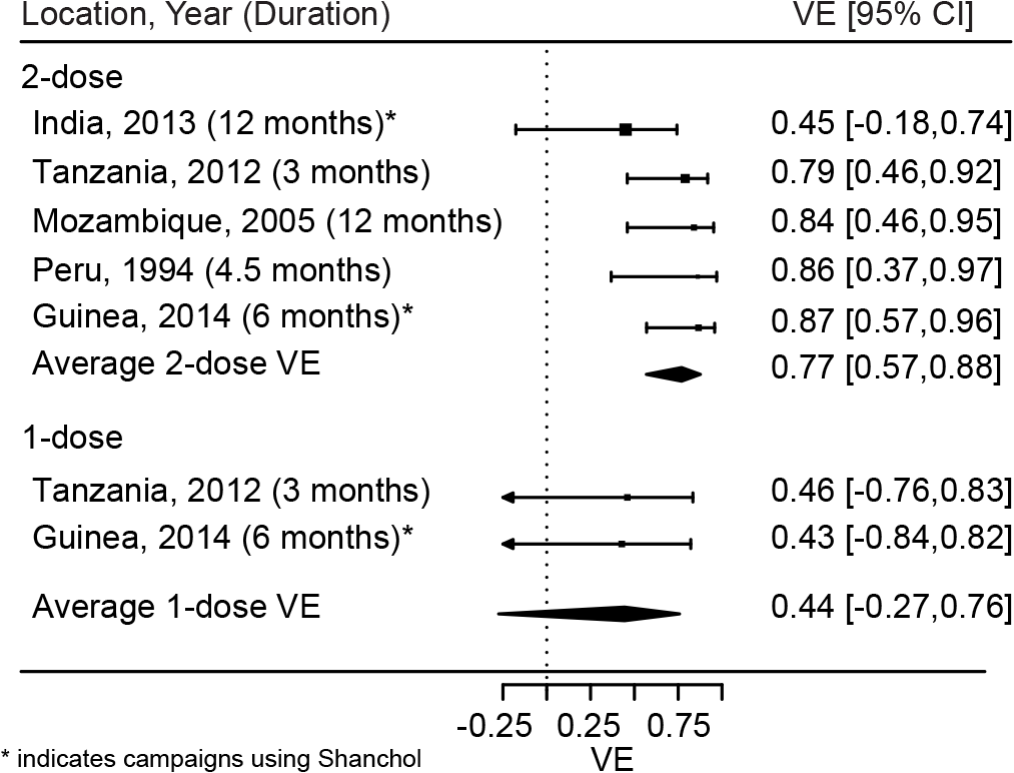
Short-term protection from one and two doses of oral cholera vaccine. Reported estimates and results from random effects regression models (filled diamonds) for both one (bottom) and two (top) doses of OCV. VE, vaccine efficacy.

Based on a meta-analysis of these five published studies, we estimate the average short-term protection (up to 1 y) conferred by two doses of killed whole cell OCV to be 77% (95% CI 57%–88%; Fig 4) [5,24–26,28]. No studies to our knowledge have specifically aimed to measure the effectiveness of one dose, but two studies have reported single-dose OCV effectiveness as a secondary outcome (Khatib et al. [24], vaccine effectiveness = 46%; Luquero et al. [5], vaccine effectiveness = 43%), suggesting a short-term vaccine effectiveness of 44% (95% CI −27% to 76%). These findings lead to an estimate of the RSE of 57% (0.44/0.77 × 100; interquartile range 13%–88%).

### Minimum Relative Single-Dose Efficacy in a Reactive Vaccination Campaign

For the simple case of a preemptively administered all-or-nothing vaccine, the same number of people would be protected by a single dose-campaign as would be protected by a two-dose campaign that was twice as efficacious (i.e., MRSE = 50%), since twice as many people would be reached. However, this simple thought experiment does not capture the effects of a potentially leaky vaccine (i.e., only partial protection conferred to vaccinee) or the dynamics of vaccination during an ongoing epidemic.

**Fig 5:**
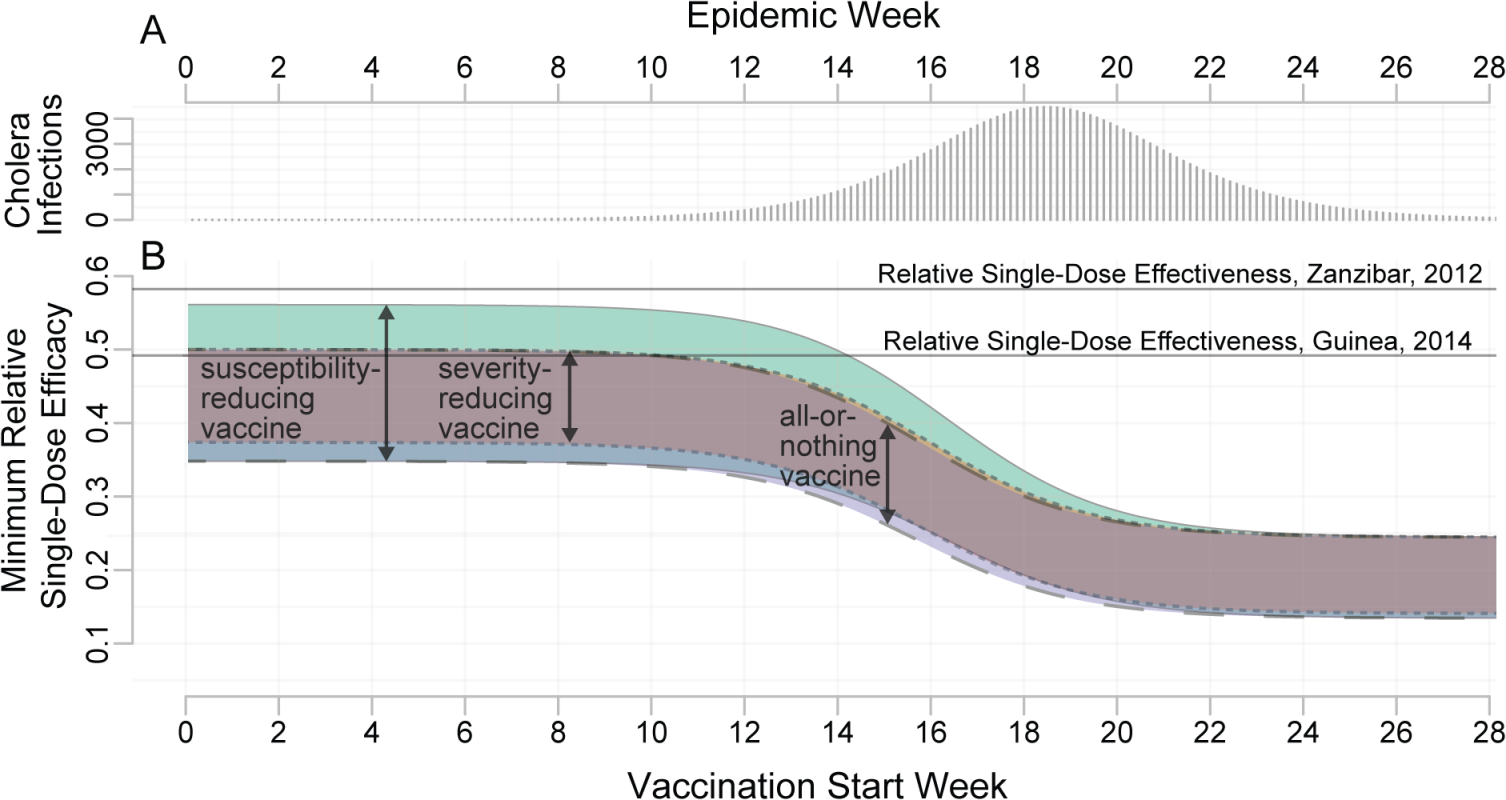
Minimum relative single-dose efficacy for oral cholera vaccine. (A) Unvaccinated epidemic calibrated to the 2008 epidemic in Bissau, Guinea-Bissau (see S2 Text). (B) MRSE needed to avert at least as many cases as a two-dose campaign with the same number of doses, for different delays in the start of the vaccination campaign (*x*-axis). The colored bands represent the range of estimates for the MRSE for different assumed vaccine mechanisms: a susceptibility-reducing vaccine (green, solid outline), a severity-reducing vaccine (orange, dotted outline), and an all-or-nothing vaccine (blue, dashed outline). The widths of the colored bands reflect the range of MRSEs resulting from varying vaccination coverage (1,000–500,000 doses in a population of 500,000). This range captures the impact of indirect protection (i.e., herd immunity) as well as direct protection. Horizontal lines represent estimates of the RSE from the literature.

The MRSE for a reactive OCV campaign depends on the timing and size of the campaign. Early in the epidemic, the MRSE ranges between 35% and 56% depending on the properties of the vaccine and percentage of the population covered (Fig 5). A single-dose campaign performs best (i.e., the MRSE is lowest) when large portions of the population can receive a single dose, maximizing the impact of early indirect protection. The worst case for a single-dose campaign (i.e., the MRSE is highest) occurs when we can reach only a small fraction of the population with a susceptibility-reducing vaccine. The relative performance of a single-dose campaign increases (i.e., the MRSE decreases) as the epidemic progresses. In our hypothetical population, during the period of epidemic growth, the MRSE fell by approximately 1% every 3 d, reaching between 17% and 32% by the epidemic peak. After the peak, the MRSE continued to fall, and reached 15% to 25% in the tail of the epidemic (Fig 5B). In general, the more explosive the epidemic, the quicker the MRSE drops as the epidemic progresses. Most of the MRSE estimates, even from vaccination early in the epidemic, fall below the two published estimates of RSE from the literature (49% [5] and 58% [24]), suggesting this threshold will often be attained.

In simulations, as the proportion of environmentally mediated (as opposed to person-to-person) transmission increased [15], the impact of vaccination delays on the MRSE decreased. When the proportion of environmental transmission is large enough, the MRSE remains constant throughout the epidemic, but never exceeds that seen in a preemptive campaign (S4 Text).

### Individual versus Population Benefit

**Fig 6:**
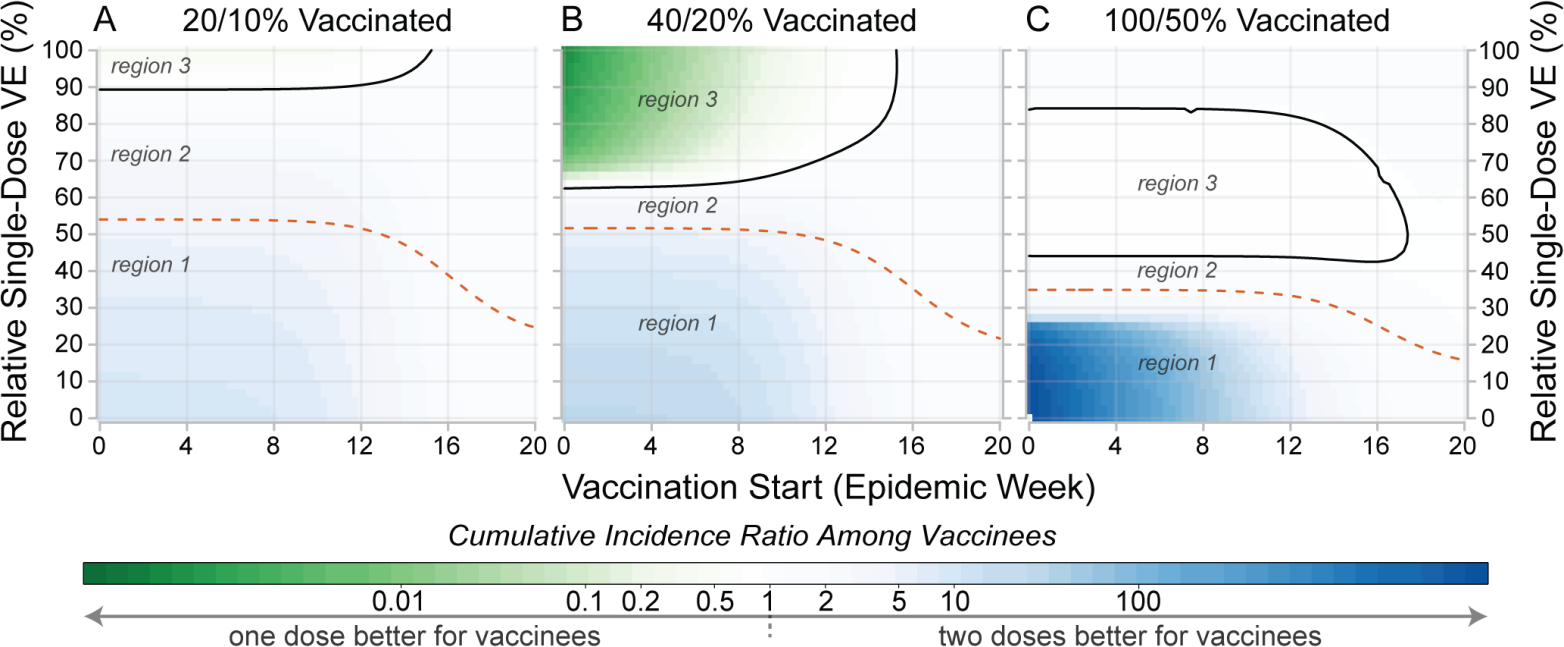
Comparison of individual- and population-level benefits of one- and two-dose campaigns by vaccination start time and relative single-dose efficacy. Colors in each panel represent the cumulative incidence ratio, comparing cumulative incidence among those ever receiving vaccine in one-dose campaigns (numerator) with the cumulative incidence among those ever receiving vaccine in two-dose campaigns (denominator). Solid lines in each panel outline the region where single-dose campaigns are better for vaccinees (a result of indirect effects). Dashed lines represent the population-level threshold above which overall cumulative incidence is lower in a one-dose campaign compared to a two-dose campaign. Panels illustrate settings where enough susceptibility-reducing vaccine is available to cover (A) 20%, (B) 40%, and (C) 100% of the population with a single dose. In each plot, region 1 corresponds to the area where two doses are better for both the vaccinees and population, region 2 is where one dose is better for the population but not the vaccinees, and region 3 is where one dose is better for vaccinees and the population. Similar plots for different forms of vaccine protection are shown in S4 Text. VE, vaccine efficacy.

Early in an epidemic, those vaccinated are sometimes better off in a two-dose campaign, even if a single-dose campaign will prevent more cases overall (green-shaded areas in Fig 6). For instance, in simulations with 200,000 doses distributed early in a population of 500,000, the MRSE was 51%; however, those receiving the vaccine were better off (i.e., had a lower risk of infection) in a single-dose campaign only if a single dose was at least 62% as efficacious as the two-dose protocol (Fig 6B). In general, both vaccination timing and coverage play a large role in determining the gap in the RSE thresholds for individual- and population-level benefits (i.e., the gap between the dashed and solid lines in Fig 6). When vaccine coverage is low, vaccinees will often experience higher risk in a one-dose scenario than they would in a two-dose scenario unless the one-dose efficacy is much higher than the MRSE (Fig 6A). As vaccination is delayed towards the end of the epidemic, the impact of both vaccination strategies diminishes, and while one-dose campaigns may avert more cases, the risk of disease within vaccinees, though low, is relatively higher than it would be if they received two doses.

### Performance of Hypothetical Single-Dose Campaigns in Zimbabwe, Haiti, and Guinea

**Fig 7:**
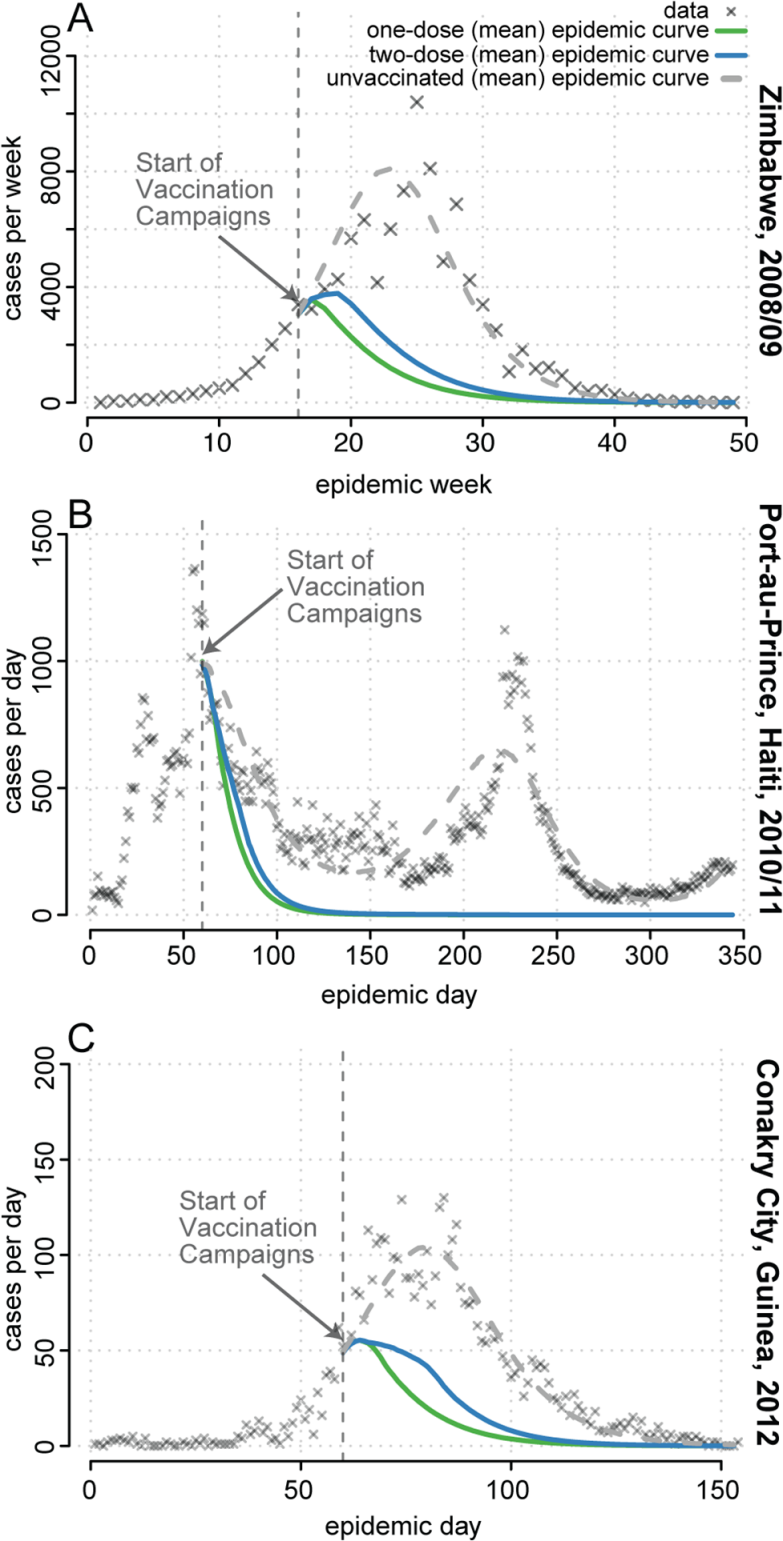
Mean projected epidemic trajectories from simulated one- and two-dose reactive vaccination campaigns compared to observed epidemics. (A) Zimbabwe. (B) Port-au-Prince, Haiti. (C) Conakry, Guinea. Simulated campaigns had enough vaccine to cover 50% of the population with a single dose of a severity-reducing vaccine. Shown are reported cholera cases (grey X’s), the mean number of cases at each time point in simulated epidemics with no vaccination (dashed grey line), simulated epidemics with a two-dose campaign (blue line), and simulated epidemics with a single-dose campaign (green line). See S3 Text for details and additional results.

The 2008–2009 cholera outbreak in Zimbabwe resulted in 98,591 reported cholera cases and 4,288 deaths (Fig 7A) [20], with an estimated basic reproductive number (*R*_0_) of 1.2 based on our model (S3 Text). OCV was not used in the outbreak response. If vaccination had begun 4 mo after the first reported case, once control activities were fully underway, with enough vaccine to cover 50% of the population with one dose (i.e., 6.7 million doses), a single-dose campaign would have averted an estimated 70,584 cases (95% prediction interval [PI] 55,943–86,205) and 2,998 deaths (95% PI 2,320–3,720). This one-dose campaign averted 1.16 times (95% PI 1.03–1.33) as many cases in simulation as a two-dose campaign with the same quantity of vaccine (Fig 7A).

Cholera struck Haiti in October 2010 and continues to circulate. The first two waves of the epidemic resulted in an estimated 119,902 cases in Port-au-Prince [21], the capital (Fig 7B), where we estimated *R*_0_ to be 1.2 at the start of the epidemic (S3 Text). After initial rejection of vaccine due to supply constraints and logistical complexity, vaccination was revisited by the Pan American Health Organization in mid-December 2010 [29]. If vaccination had started when revisited, with enough vaccine to cover 50% of the Port-au-Prince population with a single dose (i.e., 1,050,000 doses), a one-dose campaign would have averted 78,317 (95% PI 57,435–100,150) cholera cases and 783 deaths (95% PI 574–1002), assuming a 1% case fatality ratio [4]), 1.05 times (95% PI 1.01–1.10) as many cases and deaths as a two-dose campaign (Fig 7B). This relatively small, but significant, difference between the strategies is primarily due to vaccination beginning when the reproductive number is near its (seasonal) minimum, thus allowing both campaigns to rapidly halt transmission.

A two-dose OCV campaign was conducted in Guinea in response to the 2013 cholera epidemic in two remote rural areas but not in the capital city, Conakry [18], where we estimated *R*_0_ to be 3.8 (S3 Text). If an equally timely reactive campaign had taken place in Conakry, starting 2 mo after the first case was reported, we estimate a single-dose campaign covering 50% of the population (i.e., 828,150 doses) would have averted 2,826 (95% PI 2,490–3,170) of the 4,566 reported clinical cholera cases and 51 deaths (95% PI 45–57), 1.21 times (95% PI 1.11–1.32) as many cases and deaths as a two dose campaign (Fig 7C).

In sensitivity analyses we explored the potential outcomes of campaigns using alternative vaccination times and vaccine efficacies (S3 Text) and found our qualitative results to hold. As vaccination is delayed, the overall impact in these epidemics of both one- and two-dose campaigns diminishes, although the relative impact of one dose generally increases. When the RSE was reduced to half the value used in the main analysis (28% RSE and 22% vaccine efficacy)—a distinct possibility, particularly in populations previously unexposed to cholera—simulated single-dose campaigns during historic epidemics no longer averted significantly more cases or deaths than two-dose campaigns. Though the presented model was best supported by the data, concerns remain that the amount of data available for Haiti is inadequate to rule out alternative models. Hence, we considered five alternative models and found that the relative impact of one versus two doses remained nearly identical (though the 95% PIs included 1.0 in three of the five models; S3 Text).

## Discussion

In the midst of a rapidly growing cholera epidemic, swift action pays dividends. Our results suggest that providing a less protective single-dose OCV regimen to more people could have larger public health benefits than providing the recommended two-dose schedule to fewer people. In the face of inevitable delays, the short-term benefits of rapid action are even greater, and the single-dose efficacy needed to make a single-dose OCV campaign have at least the public health impact of a two-dose campaign falls to levels well below current (albeit highly uncertain) estimates. The costs and logistical challenges of multi-dose vaccine campaigns are substantial, particularly during the humanitarian emergencies that often presage cholera epidemics. If one-dose campaigns are no less effective than their two-dose counterparts, their simplicity may make them the preferred choice in many settings.

Our results contribute to a growing body of evidence, based on both empirical and computational studies, providing the basis for effective strategies for OCV use in outbreaks [5,18,20,30–32]. We present a novel framework for weighing the public health utility of using a single dose in reactive OCV campaigns in light of current and future estimates of the efficacy of this alternative regimen. Our results highlight the trade-offs between population- and individual-level benefits of OCV dosing strategies, an important though rarely discussed topic.

In areas with epidemic cholera, outbreaks often recur within 5 y [2,33]. Hence, the long-term individual and public health benefits of vaccination should also be considered when deciding between one- and two-dose campaigns. Little is known about the long-term efficacy of one OCV dose across epidemiologic settings, and even the persistence of two-dose immunity is uncertain in settings where cholera exposure is rare. Conducting a single-dose campaign does not necessarily preclude the use of a second dose once more supplies become available. Follow-up campaigns should be considered after a single-dose reactive campaign; however, limited data exist on the efficacy of OCV vaccination with longer inter-dose periods.

The evidence for the efficacy of a single dose of OCV is mixed. Field trials and immunogenicity studies of Shanchol suggest a moderate protective effect [5,24,34]. An early field trial with a related vaccine in Bangladesh showed no evidence of protection from a single dose [35]. However, Shanchol has a higher antigen concentration than the vaccine used in that trial and has been shown to illicit a more robust immune response (as measured by vibriocidal antibodies) [36,37]. The same trial suggested that the addition of the B subunit of the cholera toxin may enhance protection in the first 6 mo after vaccination, and the subunit is included in the other internationally licensed OCV, Dukoral [38]. Unfortunately, addition of the B subunit requires that the vaccine be administered with a buffer, increasing the logistical complexity and costs. In addition to vaccine properties, different host characteristics may affect the efficacy of one dose of OCV. A single dose of OCV may act as a booster of the immune system in populations where cholera regularly circulates. It is unclear whether immunologically naïve vaccinees (like most in Haiti before the first wave of cholera) will benefit from the same level of protection. Similarly, a single dose of OCV may have a differential effect in children and adults, because of both differences in their immune response and differences in historical exposure [34].

The decision of how to use a limited vaccine supply ultimately depends on the goals of a vaccine campaign. Here we focused on vaccination in large populations where minimizing the total number of cholera cases within an epidemic was the goal. However, urban populations, like that of Conakry, are often the easiest to provide with supportive care as well as water and sanitation interventions. Consequently, case fatality rates may be highest in remote areas and in vulnerable populations, and in these populations OCV may have the greatest impact on mortality. Vaccine supply may not be limited compared to the size of these groups, and “reactive” campaigns might be conducted even before an epidemic if triggered by the appearance of cholera in distant urban centers. This may change the one- versus two-dose calculus, particularly if these groups are likely to remain at risk of poor outcomes in future outbreaks.

Likewise, some may feel that even a very safe medical intervention like OCV must first aim to provide the maximum benefit to those receiving the intervention, regardless of public health concerns [25,39,40]. However, our results show that in many realistic reactive settings a single-dose campaign will also often maximize the benefits to those receiving vaccine over the short term. In certain settings with low vaccine coverage, vaccinees may experience higher risk receiving a single dose than they otherwise would have with two doses. Whether this is acceptable and whether these individuals should consent to receiving an off-label regimen are both ethical issues that public health officials may have to confront when making allocation decisions.

At times vaccine supply may not be the dominant rate-limiting factor driving the decision of whether to use a one- or two-dose regimen. In settings where future access may be uncertain (e.g., areas cut off from transport during the rainy season or situated in a conflict zone), a single dose will most likely be preferred. In other settings, cost-effectiveness estimates may drive the decision, and there may be cases where the additional costs, time, and effort incurred by these rate-limiting factors make a single dose more cost-effective even if two doses are projected to have a larger impact. Furthermore, the use of a less efficacious single-dose regimen could lead to perceptions that the vaccine is ineffective, ultimately leading to lower acceptance by communities and public health decision-makers. However, our analysis shows that in many cases fewer people receiving the vaccine get cholera in the one-dose versus the two-dose scenario (at least within a single epidemic), and an ineffective two-dose campaign could also negatively impact perceptions. To fully explore these trade-offs in a particular setting, more detailed site-specific data are required.

As with any study evaluating a hypothetical intervention, this study has numerous limitations. Our models and results are based on direct vaccine efficacy, but trials (except challenge studies) provide some measure of vaccine effectiveness, rather than vaccine efficacy, in populations. Hence, our estimates of vaccine effectiveness may not precisely capture relative efficacy; however, three of the five studies considered were designed to estimate vaccine effectiveness in such a way as to approximate efficacy [5,18,28]. The models used are simplifications of the true transmission process, and the epidemiology of cholera may differ markedly across settings. In particular, we assume homogenous cholera risk across large populations, while, in reality, many will not be at risk for infection. The efficiency of cholera transmission and the relative importance of different mechanisms of spread will also differ between settings. While our estimates of the basic reproductive number of 1.2 in the Zimbabwe and Haiti outbreaks are consistent with previous analyses [41,42] (no previous estimates published for the Conakry outbreak), the homogeneity assumptions used in our models may lead to underestimation of uncertainty in model parameters and in some cases biased estimates. If one dose is significantly less efficacious than our current best estimates (perhaps in cholera-naïve populations, where efficacy is uncertain), our predictions of the cases and deaths averted in historical epidemics may be overestimates. However, as shown in sensitivity analyses (S3 Text), even a very low efficacy single dose (22% vaccine efficacy) could have averted approximately the same number of cases as a hypothetical two-dose campaign. Finally, we did not consider models with transmission from autochthonous environmental vibrios. If a significant proportion of cases were a result of this transmission mechanism, indirect protection from the vaccine would be diminished, and the trade-offs between one and two doses would change. Our main results capture much, but not all, of this uncertainty in cholera epidemiology and vaccine effects, and extensive sensitivity analyses did not qualitatively change the results (S4 Text).

Despite renewed interest in OCV, supplies are likely to remain limited for years to come. As illustrated by recent cholera outbreaks in Haiti, West Africa, and South Sudan, the demand for cholera control tools seems unlikely to decrease any time soon. In light of this situation, the public health community must ask itself how best to use limited resources, and what evidence is needed before innovative strategies—many differing from the internationally licensed protocol—are tried. Our analysis shows that there is a low bar for single-dose campaigns, with one dose of vaccine not needing to be more than 50% as efficacious (and perhaps much less) as two doses for a single dose to be preferred in reactive campaigns. Current evidence suggests that a single dose of Shanchol may meet this threshold [5,24,34,36]. However, substantial uncertainty about one-dose efficacy remains, and field studies in areas with periodic outbreaks combined with careful evaluation of any one-dose campaigns should remain a priority. Through the efforts of the global health community, one day OCV may no longer be a limited resource, and perhaps an even more effective field-adapted single-dose vaccine will become available. Until then, strategies that balance short- and long-term benefits of vaccination should be considered to best use current limited vaccine supplies.

## Supporting Information

S1 Fig Illustration of vaccination campaigns used in simulations. The grey line shows the unvaccinated epidemic. The green line illustrates the epidemic curve with a single-dose vaccination campaign with 10 d of vaccination. The blue line shows the epidemic curve for a two-dose vaccination campaign with each dose administered over a 5-d period with 14 d in between.
(EPS)

S1 Text Introduction to minimum relative single-dose efficacy. (PDF)

S2 Text Overview of transmission models. (PDF)

S3 Text Overview of stochastic models and fit. (PDF)

S4 Text Sensitivity analyses. (PDF)

## Abbreviations

MRSE: minimum relative single-dose efficacy
OCV: oral cholera vaccine
PI: prediction interval
RSE: relative single-dose efficacy
