# Correction: The Impact of a One-Dose versus Two-Dose Oral Cholera Vaccine Regimen in Outbreak Settings: A Modeling Study

**DOI:** 10.1371/journal.pmed.1001989

**Published:** 2016-03-11

**Authors:** Andrew S. Azman, Francisco J. Luquero, Iza Ciglenecki, Rebecca F. Grais, David A. Sack, Justin Lessler

Fig 4 is incorrect. The duration stated for the 2-dose effectiveness study in Tanzania (2012) should be 15 months, rather than 3 months. The authors have provided a corrected version here.

**Fig 4:**
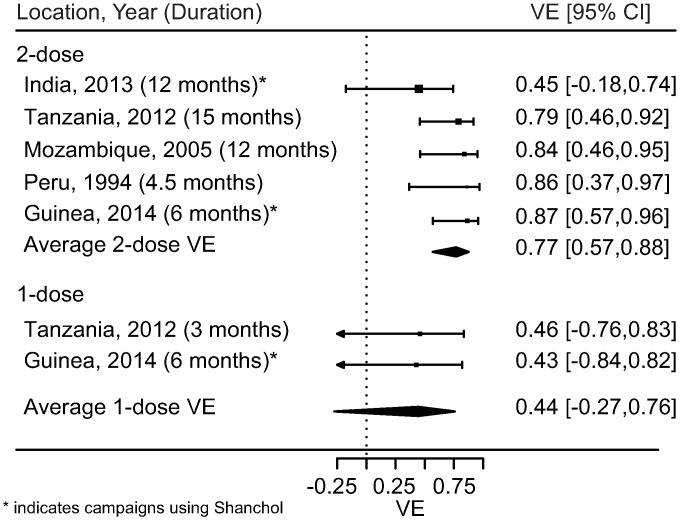
Short-term protection from one and two doses of oral cholera vaccine. Reported estimates and results from random effects regression models (filled diamonds) for both one (bottom) and two (top) doses of OCV. VE, vaccine efficacy.
