# Supplementary material for: The Impact of a One-Dose versus Two-Dose Oral Cholera Vaccine Regimen in Outbreak Settings: A Modeling Study
Source: PLoS Med. 2015 Aug 25;12(8):e1001867. doi: 10.1371/journal.pmed.1001867 (PMC4549326; doi:10.1371/journal.pmed.1001867)
Supplement: S1 Text — (PDF) [file pmed.1001867.s002.pdf]

Performance of One- versus Two-Dose Oral Cholera Vaccine Campaigns in Response to Outbreaks
S1 Text: Overview of Transmission Models

Andrew S. Azman, Francisco J. Luquero, Iza Ciglenecki, Rebecca F. Grais, David A. Sack, and Justin Lessler

We built a set of Susceptible Exposed Infectious Recovered (SEIR) models to encompass different aspects of cholera transmission and ways in which OCV may protect individuals. The most general model is the 'two-path' model which allows for both person-to-person and environmentally mediated transmission and other models include only a single transmission pathway. Parameters shared by all models are displayed in Table S1-1 and additional parameters are shown in the subsections below. In the deterministic versions used in the main text, the transmission parameter β was fit (through minimization of the squared residuals) to the observed daily case reports from a 2008/9 epidemic in Bissau City, Guinea Bissau.[1]

Table S1-1: Core parameters used in deterministic transmission models

Parameter	Desc.	Value	Source
$1\backslash\sigma$	Mean latent period (assumed equal to incubation period)	$1.41\ days$	[2]
$1\backslash\gamma$	Mean duration of infectiousness	$2.0\ days$	[3]
ρ_1	Vaccination rate for first dose	varied	[4]
ρ_2	Vaccination rate for first dose	varied	[4]
β	Transmission parameter	$0.654\ days^{-1}$	calibrated
θ_1	1-dose vaccine efficacy	varied	meta-analysis
θ_2	2-dose vaccine efficacy	varied	meta-analysis

All-or-Nothing Vaccination Model

With all-or-nothing vaccination θ_1 (i.e. VE) of the individuals vaccinated with dose 1 are expected to be 100% protected from infection. In this two-dose all-or-nothing model, we create states for unvaccinated (subscript 0), single-dose vaccinated (subscript 1), two-dose vaccinated (subscript 2). Only those individuals who have received a first dose are at risk of receiving a second dose. With the second vaccination, $\frac{1-\theta_2}{1-\theta_1}$ of those unprotected from the first dose (S_1) remain unprotected moving to S_2. The additional individuals protected per second dose given to an unprotected first dose recipient is:

$$\text{additional protected with second dose} = \overbrace{\frac{\theta_2}{1-\theta_1}}^{\text{total protected after 2-doses}} - \underbrace{\frac{\theta_1}{1-\theta_1}}_{\text{total protected after 1-dose}} \tag{1}$$

$$= \frac{\theta_2-\theta_1}{(1-\theta_1)} \tag{2}$$

This model can be described by the following system of differential equations:

$$
\begin{aligned}
N_i =& S_i + E_i + I_i + R_i & i \in (0,1,2) \quad (3)\\
\lambda =& \frac{\beta\left(I_0 + I_1 + I_2\right)}{\sum_{i\in(0,1,2)} N_i} & (4)\\
\frac{dS_0}{dt} =& -\lambda S_0 - \rho_1(t)\frac{S_0}{N_0} & (5)\\
\frac{dS_1}{dt} =& -\lambda S_1 + (1-\theta_1)\rho_1(t)\frac{S_0}{N_0} - \rho_2(t)\frac{S_1}{N_1} & (6)\\
\frac{dS_2}{dt} =& -\lambda S_2 + \frac{1-\theta_2}{1-\theta_1}\rho_2(t)\frac{S_1}{N_1} & (7)\\
\frac{dE_0}{dt} =& \lambda S_0 - \sigma E_0 - \rho_1(t)\frac{E_0}{N_0} & (8)\\
\frac{dE_1}{dt} =& \lambda S_1 - \sigma E_1 - \rho_2\frac{E_1}{N_1} + \rho_1(t)\frac{E_0}{N_0} & (9)\\
\frac{dE_2}{dt} =& \lambda S_2 - \sigma E_2 + \rho_2(t)\frac{E_1}{N_1} & (10)\\
\frac{dI_0}{dt} =& \sigma E_0 - \rho_1(t)\frac{I_0}{N_0} - \gamma I_0 & (11)\\
\frac{dI_1}{dt} =& \sigma E_1 - \rho_2(t)\frac{I_1}{N_1} - \gamma I_1 + \rho_1(t)\frac{I_0}{N_0} & (12)\\
\frac{dI_2}{dt} =& \sigma E_2 + \rho_2(t)\frac{I_1}{N_1} - \gamma I_2 & (13)\\
\frac{dR_0}{dt} =& \gamma I_0 - \rho_1(t)\frac{R_0}{N_0} & (14)\\
\frac{dR_1}{dt} =& \gamma I_1 + \theta_1\rho_1(t)\frac{S_0}{N_0} + \rho_1(t)\frac{R_0}{N_0} - \rho_2(t)\frac{R_1}{N_1} & (15)\\
\frac{dR_2}{dt} =& \gamma I_2 + \frac{\theta_2-\theta_1}{1-\theta_1}\rho_2(t)\frac{S_1}{N_1} + \rho_2(t)\frac{R_1}{N_1} & (16)
\end{aligned}
$$

Susceptibility-Reducing Vaccine Model (VE_S)

Our first leaky vaccine model (VE_S) reduces the risk of infection by $\theta_.$ in all vaccinees. Figure S1-1 illustrates the model structure and flows between states; with circles representing states and edges representing rates of transition from one state to another.

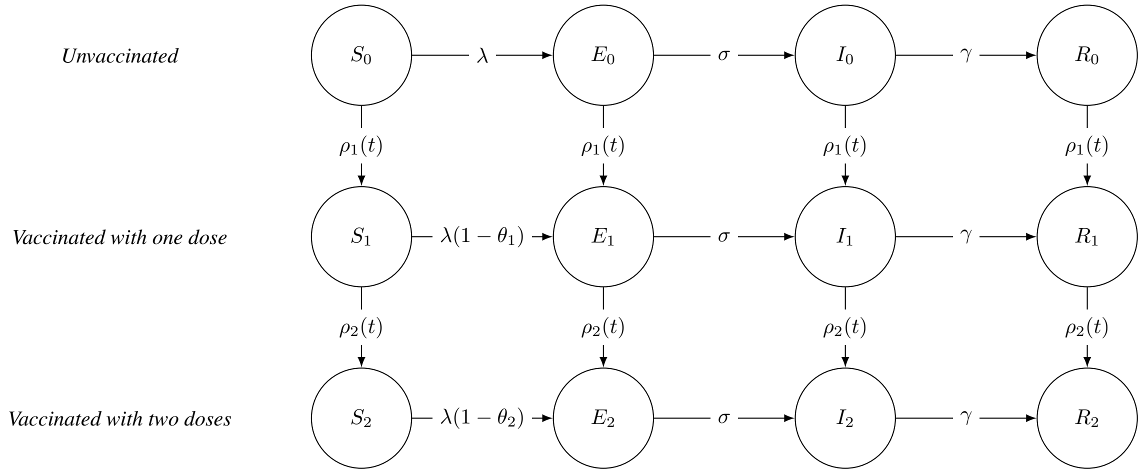

Figure S1-1: Flow diagram of susceptibility-reducing vaccine model VE_S Model

The following system of equations describes the VE_S vaccine model:

$$N_i = S_i + E_i + I_i + R_i \qquad i \in (0, 1, 2) \tag{17}$$

$$\lambda = \frac{\beta}{\sum_{i=0,1,2} N} (I_0 + I_1 + I_2) \tag{18}$$

$$\frac{dS_0}{dt} = -\lambda S_0 - \rho_1(t)\frac{S_0}{N_0} \tag{19}$$

$$\frac{dS_1}{dt} = -\lambda(1-\theta_1)S_1 + \rho_1(t)\frac{S_0}{N_0} - \rho_2(t)\frac{S_1}{N_1} \tag{20}$$

$$\frac{dS_2}{dt} = -\lambda(1-\theta_2)S_2 + \rho_2(t)\frac{S_1}{N_1} \tag{21}$$

$$\frac{dE_0}{dt} = \lambda S_0 - \rho_1(t)\frac{E_0}{N_0} - \sigma E_0 \tag{22}$$

$$\frac{dE_1}{dt} = \lambda S_1(1-\theta_1) + \rho_1(t)\frac{E_0}{N_0} - \rho_2(t)\frac{E_1}{N_1} - \sigma E_1 \tag{23}$$

$$\frac{dE_2}{dt} = \lambda S_2(1-\theta_2) + \rho_2(t)\frac{E_1}{N_1} - \sigma E_2 \tag{24}$$

$$\frac{dI_0}{dt} = \sigma E_0 - \rho_1\frac{I_0}{N_0} - \gamma I_0 \tag{25}$$

$$\frac{dI_1}{dt} = \sigma E_1 + \rho_1\frac{I}{N_0} - \rho_2(t)\frac{I_1}{N_1} - \gamma I_1 \tag{26}$$

$$\frac{dI_2}{dt} = \sigma E_2 + \rho_2(t)\frac{I_1}{N_1} - \gamma I_2 \tag{27}$$

$$\frac{dR_0}{dt} = \gamma I_0 - \rho_1(t)\frac{R_0}{N_0} \tag{28}$$

$$\frac{dR_1}{dt} = \gamma I_1 + \rho_1\frac{R}{N_0} - \rho_2(t)\frac{I_1}{N_1} \tag{29}$$

$$\frac{dR_2}{dt} = \gamma I_2 + \rho_2(t)\frac{R_1}{N_1} \tag{30}$$

Severity-Reducing Vaccine Model (VE_{SP})

The second leaky model considered reduces the probability $(1 - \theta.)$ of an individual progressing to severe symptomatic disease required the addition of a mildly-symptomatic/asymptomatic class (A). This model is described by the system of ordinary differential equations below and additional parameters are shown in Table S1-2.

Table S1-2: Additional parameters for VE_{SP} model

Parameter	Desc.	Value	Source
θ_0	Probability of asymptomatic infection without OCV	0	assumed
κ	Reduced infectiousness for asymptomatic/mildly symptomatic	0.9	assumed

The following system of equations describes the leaky severity-reducing vaccine model:

$$N_i = S_i + E_i + I_i + A_i + R_i \qquad i \in (0,1,2) \tag{31}$$

$$\lambda = \frac{\beta}{\sum_{i=0,1,2} N} \left(I + I_1 + I_2 + (1-\kappa)(A_1 + A_2)\right) \tag{32}$$

$$\frac{dS_0}{dt} = -\lambda S_0 - \rho_1(t)\frac{S_0}{N_0} \tag{33}$$

$$\frac{dS_1}{dt} = -\lambda S_1 + \rho_1(t)\frac{S_0}{N_0} - \rho_2(t)\frac{S_1}{N_1} \tag{34}$$

$$\frac{dS_2}{dt} = -\lambda S_2 + \rho_2(t)\frac{S_1}{N_1} \tag{35}$$

$$\frac{dE_0}{dt} = \lambda S_0 - \rho_1(t)\frac{E_0}{N_0} - \sigma E_0 \tag{36}$$

$$\frac{dE_1}{dt} = \lambda S_1 + \rho_1(t)\frac{E_0}{N_0} - \rho_2(t)\frac{E_1}{N_1} - \sigma E_1 \tag{37}$$

$$\frac{dE_2}{dt} = \lambda S_2 + \rho_2(t)\frac{E_1}{N_1} - \sigma E_2 \tag{38}$$

$$\frac{dI_0}{dt} = (1-\theta_0)\sigma E_0 - \rho_1(t)\frac{I_0}{N_0} - \gamma I_0 \tag{39}$$

$$\frac{dI_1}{dt} = (1-\theta_1)\sigma E_1 + \rho_1(t)\frac{I_0}{N_0} - \rho_2(t)\frac{I_1}{N_1} - \gamma I_1 \tag{40}$$

$$\frac{dI_2}{dt} = (1-\theta_2)\sigma E_2 + \rho_2(t)\frac{I_1}{N_1} - \gamma I_2 \tag{41}$$

$$\frac{dA_0}{dt} = \theta_0 \sigma E_0 - \rho_1(t)\frac{A_0}{N_0} - \gamma A_0 \tag{42}$$

$$\frac{dA_1}{dt} = \theta_1 \sigma E_1 + \rho_1(t)\frac{A_0}{N_0} - \rho_2(t)\frac{A_1}{N_1} - \gamma A_1 \tag{43}$$

$$\frac{dA_2}{dt} = \theta_2 \sigma E_2 + \rho_2(t)\frac{A_1}{N_1} - \gamma A_2 \tag{44}$$

$$\frac{dR_0}{dt} = \gamma(I_0 + A_0) - \rho_1(t)\frac{R_0}{N_0} \tag{45}$$

$$\frac{dR_1}{dt} = \gamma(I_1 + A_1) + \rho_1(t)\frac{R_0}{N_0} - \rho_2(t)\frac{R_1}{N_1} \tag{46}$$

$$\frac{dR_2}{dt} = \gamma(I_2 + A_2) + \rho_2(t)\frac{R_1}{N_1} \tag{47}$$

Two-path Transmission Model

Cholera is thought to spread via two modes of transmission, a 'fast' route dominated by person-to-person transmission, and a 'slow' route where transmission is mediated through the environment.[5] The mix of these two modes help dictate the time course of the epidemic by modifying the generation time distribution (i.e. distribution of time between infector-infected pairs). In the primary analyses we consider a subset of this model where transmission is 100% fast. Here we also consider this full two-path model to explore the impact of varying contributions of environmentally mediated (slow) transmission. The slow path is conceptualized as a series of infectious compartments which leads to a gamma (Erlang) distributed infectious period (Figure S1-2). Vaccine is implemented within this model as a leaky vaccine that reduces vaccinees susceptibility to infection (VE_S).

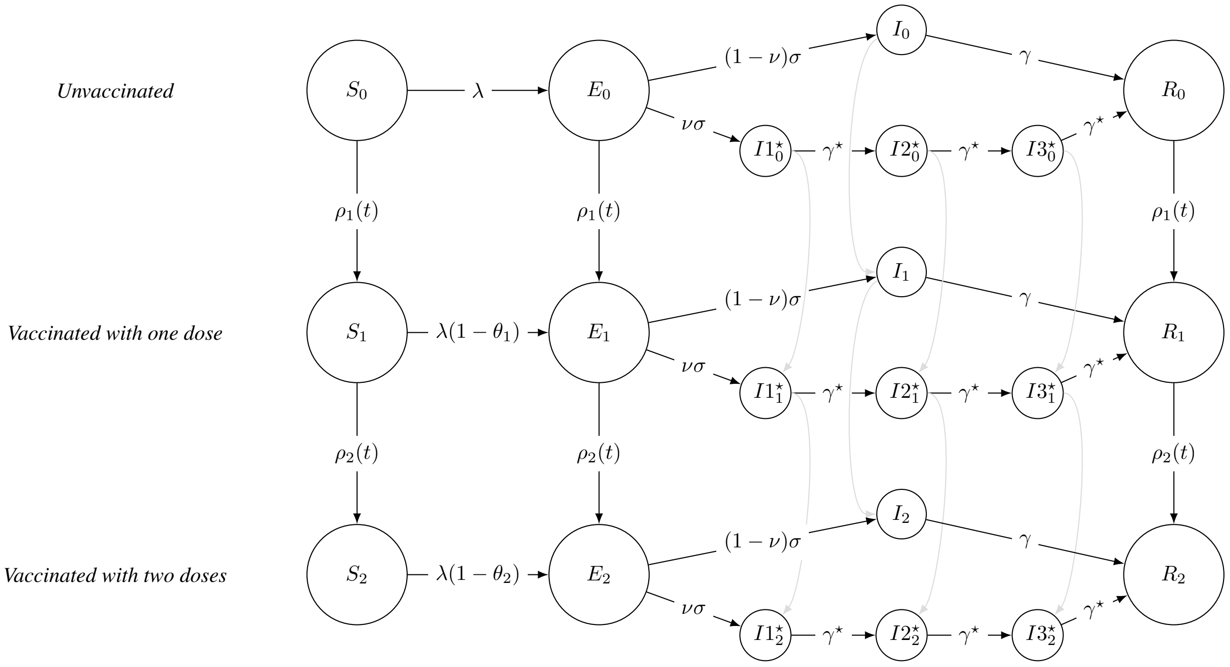

Figure S1-2: Flow diagram of two-path model. Rates from infectious compartments shown as grey edges for visualization purposes.

The infectious period distribution was fit to empirical data on the survival of *Vibrio cholerae* (Figures S1-3 and S1-4) by minimizing the squared difference between the observations and the survival function of a gamma distribution.[6] We found the best fit to include three compartments ($n_{slow} = 3$, see section Supplemental Text S4) each with a mean residence time of 7.5 days ($\gamma^\star = \frac{1}{7.5}$ See Supplemental Text S4).

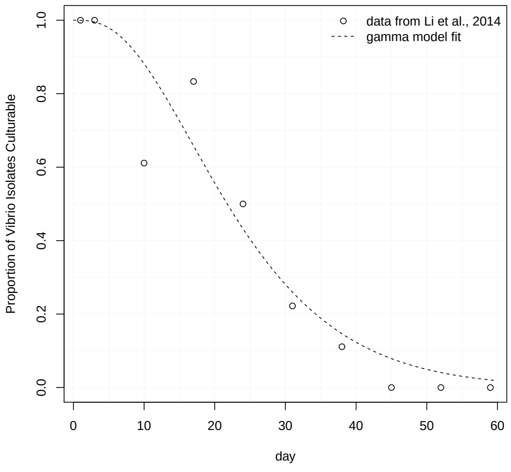

Figure S1-3: Proportion of *Vibrio cholerae* isolates surviving at different time points (from[6]) along with best fit gamma distributed survival curve.

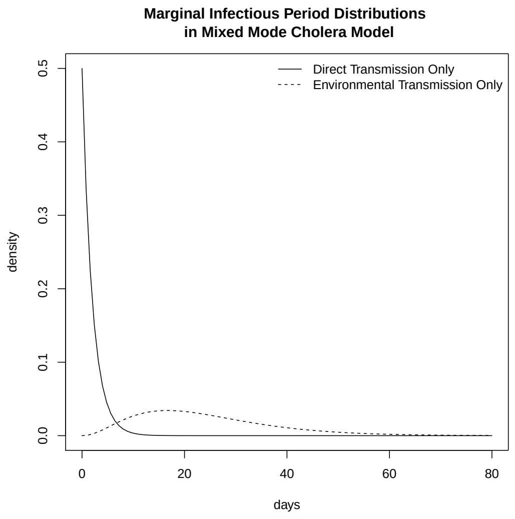

Figure S1-4: Distributions of the infectious period for the fast (person-to-person) and the slow (environmentally-mediated transmission) pathways.

References

[1] Luquero FJ, Banga CN, Remartínez D, Palma PP, Baron E, Grais RF. Cholera Epidemic in Guinea-Bissau (2008): The Importance of "Place". PloS one. 2011 May;6(5):e19005.

[2] Azman AS, Rudolph KE, Cummings DAT, Lessler J. The incubation period of cholera: a systematic review. The Journal of infection. 2013 May;66(5):432–438.

[3] Weil AA, Khan AI, Chowdhury F, LaRocque RC, Faruque ASG, Ryan ET, et al. Clinical Outcomes in Household Contacts of Patients with Cholera in Bangladesh. Clinical Infectious Diseases. 2009 Nov;49(10):1473–1479.

[4] Ciglenecki I, Sakoba K, Luquero FJ, Heile M, Itama C, Mengel M, et al. Feasibility of mass vaccination campaign with oral cholera vaccines in response to an outbreak in Guinea. PLoS Medicine. 2013;10(9):e1001512.

[5] Morris JG. Cholera–modern pandemic disease of ancient lineage. Emerging Infectious Diseases. 2011 Nov;17(11):2099–2104.

[6] Li XQ, Wang M, Deng ZA, Shen JC, Zhang XQ, Liu YF, et al. Survivability and molecular variation in Vibrio cholerae from epidemic sites in China. Epidemiology and Infection. 2014 Mar;p. 1–10.
