# Supplementary material for: The Impact of a One-Dose versus Two-Dose Oral Cholera Vaccine Regimen in Outbreak Settings: A Modeling Study
Source: PLoS Med. 2015 Aug 25;12(8):e1001867. doi: 10.1371/journal.pmed.1001867 (PMC4549326; doi:10.1371/journal.pmed.1001867)
Supplement: S2 Text — (PDF) [file pmed.1001867.s003.pdf]

Performance of One- versus Two-Dose Oral Cholera Vaccine Campaigns in Response to Outbreaks: A Modeling Study

S2 Text: Introduction to Minimum Relative Single-Dose Efficacy (MSRE)

Andrew S. Azman, Francisco J. Luquero, Iza Ciglenecki, Rebecca F. Grais, David A. Sack, and Justin Lessler

We express the main results in terms of the minimum relative single-dose efficacy, which is equal to the single-dose efficacy needed for a single-dose campaign to avert as many cases as a two-dose campaign with the same total number of doses divided by the two-dose vaccine efficacy (δ_2). Relative vaccine efficacy below this threshold will lead to fewer cases when a single-dose campaign is employed compared to a two-dose campaign with a fixed number of doses. Figure S2-1 illustrates that the MRSE depends on the two-dose vaccine effectiveness and the quantity of vaccine. We can see from Figure S2-1 (purple points) that the highest two-dose effectiveness considered (0·88, the upper limit of the 95% CI from the meta-analysis) bounds all the other MRSE estimates (i.e., the simulations with 1,000 and 500,000 doses of a vaccine with a single-dose efficacy of 0.88 bound all of the other estimates consistent with the data). Therefore, in order to capture uncertainty in the two-dose efficacy estimates, in the main text we conservatively present results from simulations using a two-dose effectiveness of 0·88 unless otherwise mentioned.

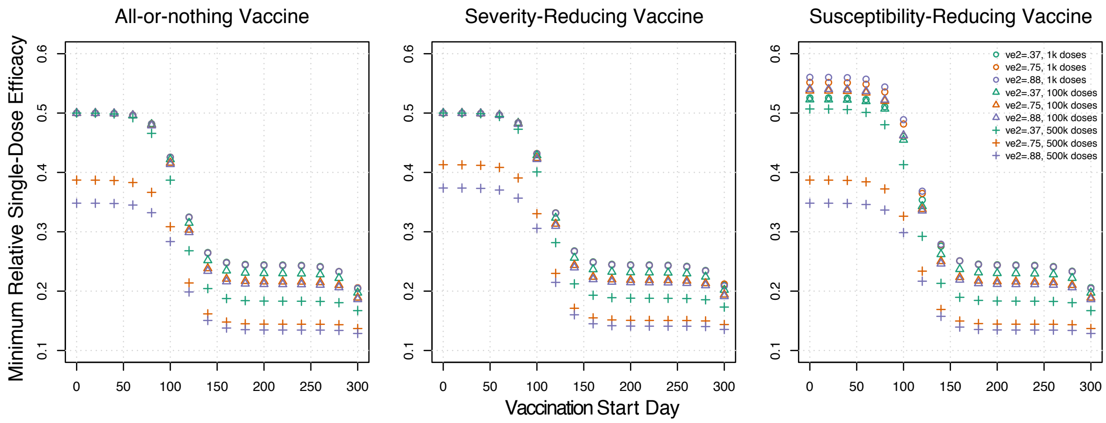

Figure S2-1: Illustration of MRSE for varying two-dose vaccine efficacies and vaccine coverage. Two-dose vaccine efficacies spanning the limits of the 95% confidence intervals from the random effects model, and doses varied between enough for 0·2% to 100% of the population in a single-dose campaign.

In a simple proactive vaccination setting where a vaccine acts by removing individuals from Susceptible to Removed ('all-or-nothing vaccine',[1]), the $MRSE$ can be derived using an adaptation of the final size equations described by Longini et al. in 1978.[2] The system below represents the final size for one ($Z_1(\infty, v_1, \delta_1)$) and two-dose $Z_2(\infty, v_2, \delta_2)$) campaigns.

$$Z_1(\infty, v_1, \delta_1) = (S_1(0) - \delta_1 v_1)\left(1 - \exp\left(-\frac{\mathcal{R}_0 Z_1(\infty, v_1, \delta_1)}{N_1}\right)\right) \quad (1)$$

$$Z_2(\infty, v_2, \delta_2) = (S_2(0) - \delta_2 v_2)\left(1 - \exp\left(-\frac{\mathcal{R}_0 Z_2(\infty, v_2, \delta_2)}{N_2}\right)\right), \quad (2)$$

where v_1 represent the number of individuals vaccinated in a single dose campaign and v_2 (individuals vaccinated in a two dose campaign) is assumed to be $v_1/2$. To estimate the MRSE, we can set $N_1 = N_2$ and $S_1 = S_2$ since they are the same population and then set $Z_1 = Z_2$ and solve for $\frac{\delta_1}{\delta_2}$ as follows:

$$(S_1(0) - \delta_2\frac{v_1}{2})\left(1 - \exp\left(-\frac{\mathcal{R}_0 Z_1}{N_1}\right)\right) = (S_1(0) - \delta_1 v_1)\left(1 - \exp\left(-\frac{\mathcal{R}_0 Z_1}{N_1}\right)\right) \quad (3)$$

$$(S_1(0) - \delta_2\frac{v_1}{2}) = (S_1(0) - \delta_1 v_1) \quad (4)$$

$$\frac{\delta_1}{\delta_2} = \frac{1}{2} \quad (5)$$

Thus in a simple all-or-nothing proactive vaccination setting, a single dose must be at least half as efficacious as two doses to avert at least as many cases over the course of the epidemic.

References

[1] Halloran ME, Struchiner CJ, Longini IM. Design and Analysis of Vaccine Studies. Springer Verlag; 2009.

[2] Longini IM, Ackerman E, Elveback LR. An optimization model for influenza A epidemics. Mathematical biosciences. 1978 Jan;38(1-2):141–157.
