# Supplementary material for: The Impact of a One-Dose versus Two-Dose Oral Cholera Vaccine Regimen in Outbreak Settings: A Modeling Study
Source: PLoS Med. 2015 Aug 25;12(8):e1001867. doi: 10.1371/journal.pmed.1001867 (PMC4549326; doi:10.1371/journal.pmed.1001867)
Supplement: S3 Text — (PDF) [file pmed.1001867.s004.pdf]

Performance of One- versus Two-Dose Oral Cholera Vaccine Campaigns in Response to Outbreaks
S3 Text: Overview of Stochastic Models and Fit

Andrew S. Azman, Francisco J. Luquero, Iza Ciglenecki, Rebecca F. Grais, David A. Sack, and Justin Lessler

We created a stochastic version of the severity reducing vaccine (VE_{SP}) model that allows for both stochasticity in the disease and the observation processes. We assumed that the transitions between states followed an Euler-Multinomial process (i.e., exponentially distributed sojourn times),[1] and that cases at time t (observations) are distributed as:

$$Cases(t) \sim NegBinom(\rho I(t), \theta)$$

where ρ is the observation fraction, θ is the dispersion parameter, and $I(t)$ is the number of infectious individuals at time t. All fitting and simulations were performed using the `pomp` package (version 0.49-2) in R.[2,3]

Zimbabwe, 2008/09

Country-wide data from the 2008-2009 cholera epidemic in Zimbabwe came from the authors of Reyburn et al.[4] Country-level data was used in this exercise due to the unavailability of adequate city-level data, as in the other illustrative examples. We fit this model with maximum-likelihood via iterated filtering (using the mif2 algorithm, an improved version of that described in Ionides et al[5]) starting at the first week of the data. We fit two parameters in this model, the transmission rate (β), and the duration of infectiousness ($1/\gamma$), and three initial conditions, the number of infectious ($I(0)$), exposed ($E(0)$), and susceptible ($S(0)$) individuals at the time the first case was observed. Other parameters were based on published literature or expert opinion. Parameter values are shown in Table S3-1.

In fitting this model we first used a trajectory matching algorithm implemented in pomp to get to values of the parameters that could come close to reproducing the observed epidemic curve. We perturbed these parameter values to get 10 sets of initial parameters and ran 10 parallel mif processes (each with 5000 particles) with a hyperbolic cooling schedule. We assessed convergence visually and started another mif process with the run with the highest log-likelihood until convergence. We started our forward simulations at week 16 of the epidemic (Figure S3-1) when vaccination was assumed to start. The state of the system at the start of simulations was estimated with a particle filter.

Table S3-1: Parameters for stochastic model fit to Zimbabwe. Note that the time unit is weeks for this model. The initial susceptible fraction of the population was calaibrated using a modified mif algorithm within the pomp package and found to be 0.85 in Zimbabwe.

Parameter	Desc.	Value (Nominal 95% CI)	Source
β	Transmission parameter	4.34 (3.29-5.03)	fit
γ	Rate of recovery from infectiousness ($week^{-1}$)	3.74 (2.81-4.81)	fit
σ	Rate of becoming infectious ($week^{-1}$)	5.00	[6]
θ	Dispersion parameter for reporting	10	assumed
ρ	Mean probability of an infection being observed	0.03	[7]

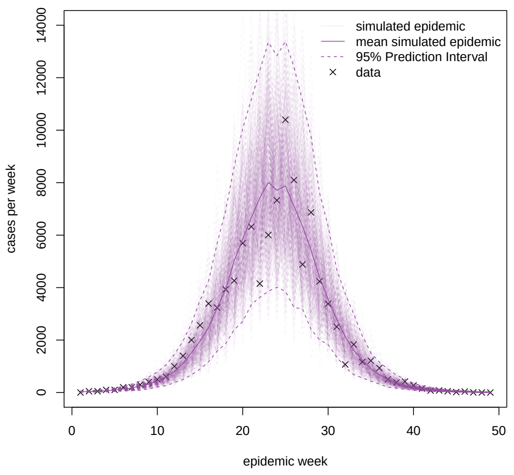

Figure S3-1: Fit of model to epidemic data (from[4]) from Zimbabwe.

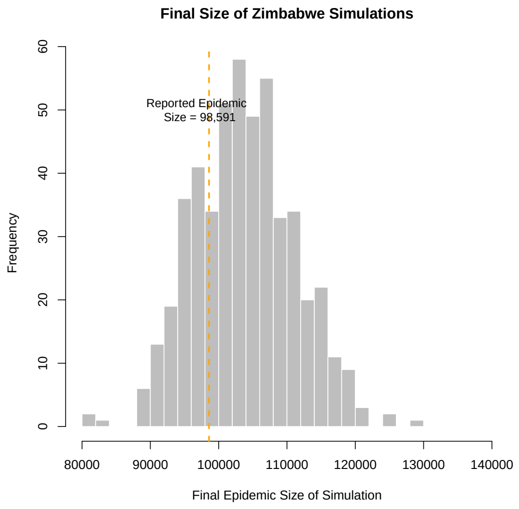

Figure S3-2: Distribution of final epidemic size of simulations compared to the reported number of cholera cases in Zimbabwe (orange line)

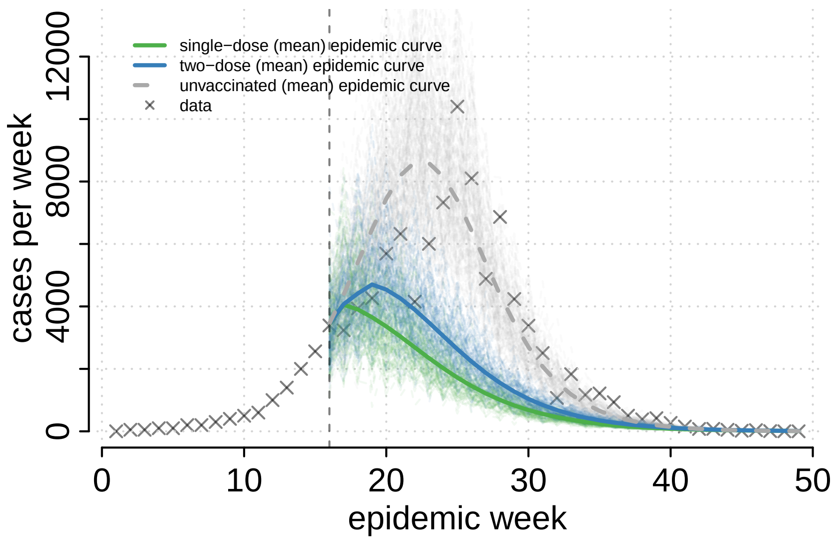

Figure S3-3: Illustration of individual simulation trajectories in unvaccinated and vaccinated (one and two-dose campaigns) scenarios in Zimbabwe. Bold lines represent the mean of simulations (as shown in the main manuscript) and lighter lines represent individual simulation trajectories.

In the main text we present vaccination simulations started on one specific day that we believed to be feasible, however, many factors contribute to when a vaccination campaign begins. Table S3-2 shows the cases averted from one and two dose campaigns with different vaccination delays to give a sense for how this the differential impacts may differ from those presented in the main text.

Table S3-2: Outcomes from one- and two-dose vaccination simulations in Zimbabwe starting at alternate times in the epidemic ranging from 6 to 26 weeks after the first case was reported.

vaccination start week	mean (95% PI) cases averted with one-dose	mean (95% PI) ratio of cases averted 1-dose to 2-dose
6	100,531 (86,432-115,759)	1.05 (1.03-1.07)
10	94,108 (79,947-109,471)	1.09 (1.06-1.13)
14	80,946 (54,068-96,494)	1.14 (1.06-1.23)
18	60,411 (35,644-76,400)	1.16 (0.97-1.35)
22	33,542 (19,356-48,149)	1.23 (0.80-1.97)
26	13,585 (4,729-22,720)	†

†In some simulations there were no cases averted in the two-dose scenario so this ratio is not calculated

Table S3-3 shows the results from simualtions where a single dose is 50% more and 50% less protective than in the main analysis. This highlights that a more protective single dose will shift the preferred strategy towards the use of one dose where as a less protective single dose does the opposite. However, even with a vaccine with 22% efficacy, we find that there is no significant difference between the cases averted with one- and two-dose regimens.

Table S3-3: Results Cases Averted with Alternative RSEs in Zimbabwe.

RSE (1-dose VE)	Mean Cases Averted (95% PI)	Ratio of Cases Averted (95% PI)
0.28 (0.22)	45,356 (28,917-62,526)	0.82 (0.63-1.01)
0.86 (0.83)	79,642 (65,063-95,509)	1.21 (1.10-1.35)

Port-au-Prince, Haiti, 2010-2011

We fit our model to suspected cholera cases (hospitalized and not hospitalized) extracted from MSPP reports (downloaded from `http://mspp.gouv.ht/site/downloads/`) from 22-October-2010 through 30-September-2011. We built upon the model used to fit Zimbabwe data, and added two additional components, a seasonal forcing function, and a constant rate of introductions of infectious cases into the city from elsewhere in the country. We added the seasonal forcing function to capture seasonal effects (some have shown it related to seasonal rainfall[8]) and, to some degree, the time-varying effects of cholera control interventions put in place. We modeled this as a periodic basis spline with 6 basis functions and 6 degrees of freedom (fitted value shown in green in Figure S3-4). We fit the components of these additional parameters within the same maximum likelihood framework as the other models using the mif algorithm. Estimated and assumed parameters are shown in Table S3-4, and results from simulations for unvaccinated scenarios are shown in Figures S3-4 and S3-5.

We started the estimation from multiple starting locations

Table S3-4: Parameters for stochastic model fit to Port au Prince, Haiti epidemic. The initial susceptible fraction of the population was calaibrated using a modified mif algorithm within the pomp package and found to be 0.97.

Parameter	Desc.	Value (Nominal 95% CI)	Source
β_1	Transmission parameter (1st component of basis spline)	0.826	fit
β_2	Transmission parameter (2nd component of basis spline)	0.569	fit
β_3	Transmission parameter (3rd component of basis spline)	0.286	fit
β_4	Transmission parameter (4th component of basis spline)	0.546	fit
β_5	Transmission parameter (5th component of basis spline)	0.633	fit
β_6	Transmission parameter (6th component of basis spline)	0.114	fit
ι	External introduction rate	2.88×10^{-12}	fit
γ	Rate of recovery from infectiousness	0.50	[9]
σ	Rate of becoming infectious	0.71	[6]
θ	Dispersion parameter for reporting	10.00	assumed
ρ	Mean probability of an infection being observed	0.91	fit

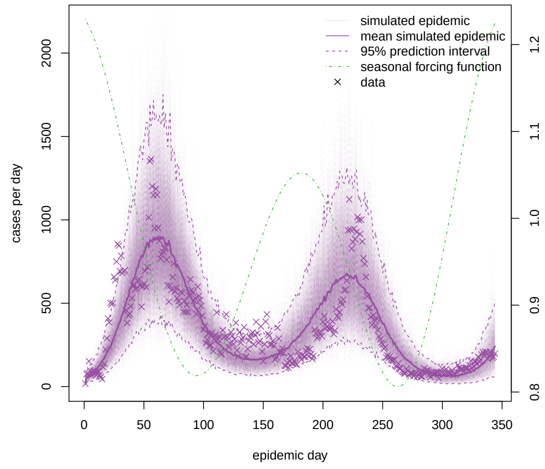

Figure S3-4: Fit of model to epidemic data from Port au Prince. Green dashed line illustrates the seasonal forcing function (secondary y-axis is the time varying basic reproductive number.)

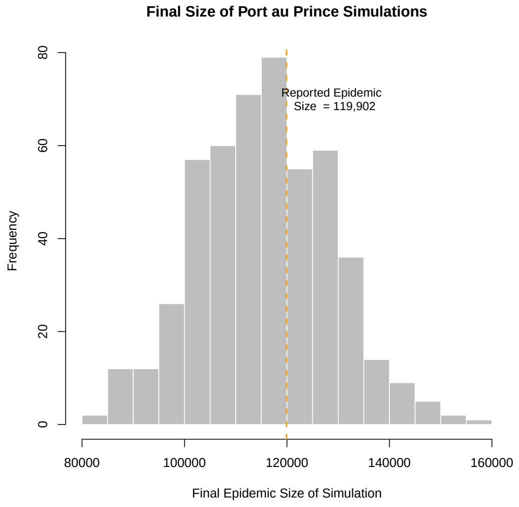

Figure S3-5: Distribution of final epidemic size of simulations compared to the reported number of cholera cases in Port au Prince (orange line)

We based our main vaccination scenario on the timing of a PAHO meeting convened to re-discuss the use of vaccine on December 17, 2010,[10] and assumed that vaccination could have started 3 days later on December 20, 2010. Epidemic trajectories from specific simulations are shown in Figure S3-6.

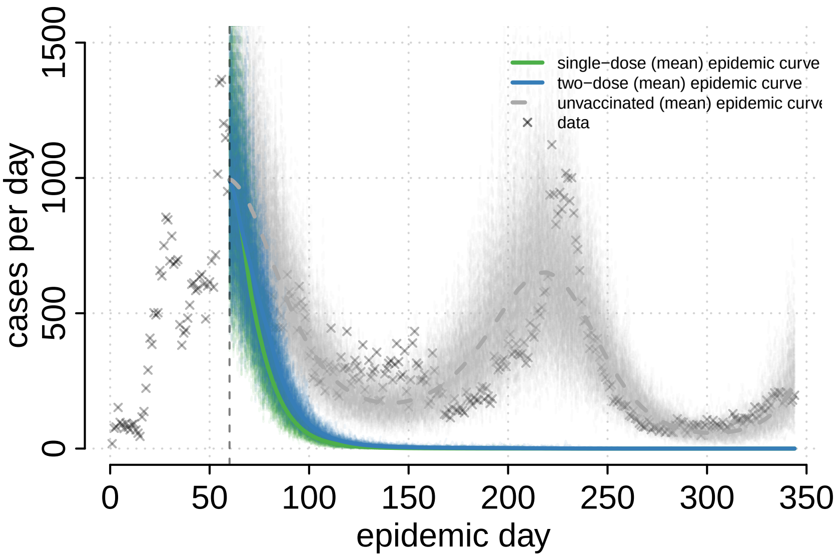

Figure S3-6: Illustration of individual simulation trajectories in unvaccinated and vaccinated (one and two-dose campaigns) scenarios in Port-au-Prince. Bold lines represent the mean of simulations (as shown in the main manuscript) and lighter lines represent individual simulation trajectories.

Table S3-5 shows the results from simualtions where a single dose is 50% more and 50% less protective than in the main analysis. This highlights that a more protective single dose will shift the preferred strategy towards the use of one dose where as a less protective single dose does the opposite. However as seen in the other modeled settings, even with a vaccine with 22% efficacy, we find that there is no significant difference between the cases averted with one- and two-dose regimens.

Table S3-5: Results Cases Averted with Alternative RSEs in Port-au-Prince.

RSE (1-dose VE)	Mean Cases Averted (95% PI)	Ratio of Cases Averted (95% PI)
0.28 (0.22)	69,815 (48,718-91,757)	0.98 (0.91-1.05)
0.86 (0.83)	82,051 (61,167-103,768)	1.06 (1.02-1.11)

With noisy data from a single epidemic, it is challenging to fit multiple co-linear parameters to the data. While we present results from the model with the highest liklihood, we explored the parameter space for alternative models and show that very similar estimates can be drawn from these models. Table S3-6 shows the next 5 best fitting model parameter sets (highest log-likelihood at the top) and the resulting estimate of the mean ratio of cases averted from 1- compared to 2-dose campaigns.

Table S3-6: Estimates of the Mean Ratio of Cases Averted with single-dose compared to two-dose campaigns for alternative models with lower log-likelihood than the main model used in the analysis (first column). Additional columns show estimates of the basic reproductive number (R_0) and model paramters for each.

	Ratio of Cases Averted (95% PI)	R_0	β_1	β_2	β_3	β_4	β_5	β_6	ι	ρ	$S(0)$
1	1.04 (1.00-1.09)	1.2	0.75	0.61	0.19	0.63	0.48	0.24	5.041e-11	0.99	0.9995
2	1.06 (1.00-1.12)	1.2	0.68	0.69	0.13	0.63	0.52	0.22	1.948e-09	0.94	0.9996
3	1.06 (1.02-1.11)	1.1	0.65	0.55	0.36	0.42	0.61	0.24	4.336e-11	0.86	0.9996
4	1.06 (0.98-1.17)	1.3	0.81	0.72	0.01	0.70	0.59	0.08	2.146e-10	0.93	0.9995
5	1.16 (1.03-1.32)	1.1	0.83	0.38	0.59	0.05	0.83	0.10	6.675e-11	0.99	0.9995

Conakry City, Guinea, 2012

We used a similar approach to fitting data from Conakry City, Guinea as we did for Zimbabwe. Due to the sputtering cases between 29-May-2012 and the end of June-2012, we fit the model starting on 30-June-2012, when the epidemic began to take off. In our vaccination scenarios we started the simulations at epidemic day 60 (27-July-2012) with the initial state of the system estimated from particle filtering of uncontrolled epidemics. Model parameters are shown in Table S3-7.

Table S3-7: Parameters for stochastic model fit to Conakry epidemic. The initial susceptible fraction of the population was calaibrated using a modified mif algorithm within the pomp package and found to be 0.30 in Guinea.

Parameter	Desc.	Value (Nominal 95% CI)	Source
β	Transmission parameter	$2.36(2.12 - 2.39)$	fit
γ	Rate of recovery from infectiousness	$0.62(0.60 - 0.68)$	fit
σ	Rate of becoming infectious	5.00	[6]
θ	Dispersion parameter for reporting	20.00	Assumed
ρ	Mean probability of an infection being observed	0.03	[7]

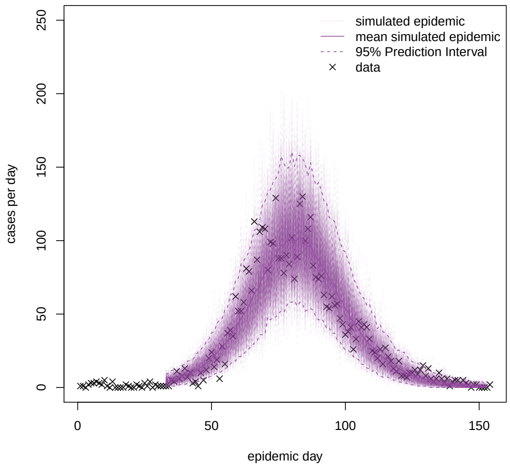

Figure S3-7: Fit of model to epidemic data from Conakry City.

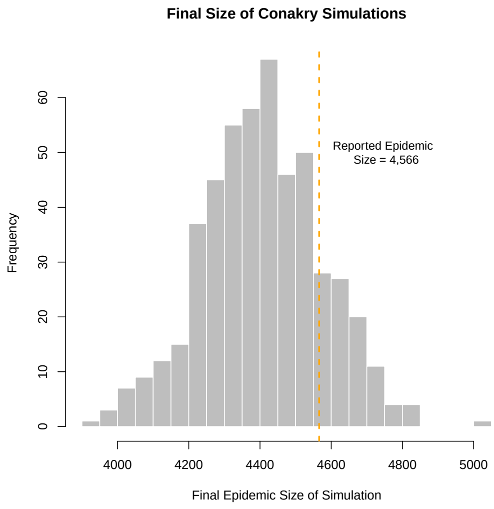

Figure S3-8: Distribution of final epidemic size of simulations compared to the reported number of cholera cases in Conakry (orange line)

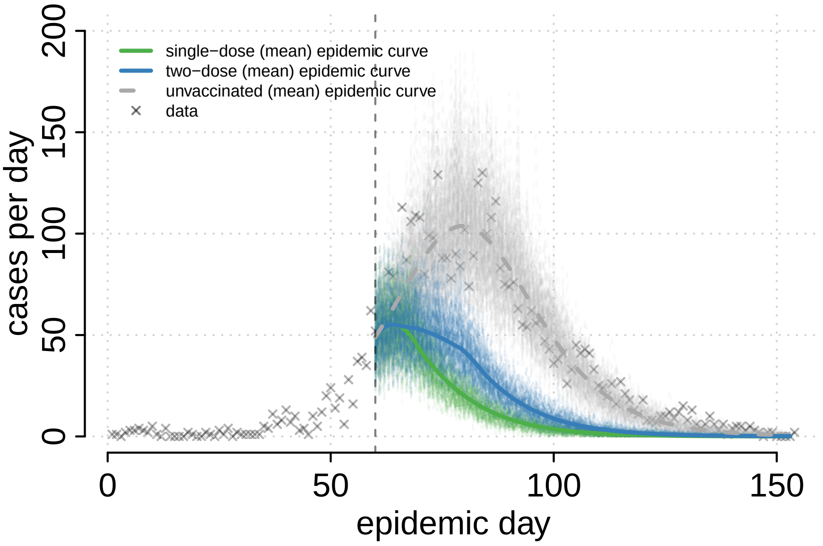

Figure S3-9: Illustration of individual simulation trajectories in unvaccinated and vaccinated (one and two-dose campaigns) scenarios in Conakry City. Bold lines represent the mean of simulations (as shown in the main manuscript) and lighter lines represent individual simulation trajectories.

In the main text we present vaccination simulations started on one specific day that we believed to be feasible, however, many factors contribute to when a vaccination campaign begins. Table S3-8 shows the cases averted from one- and two-dose campaigns with different vaccination delays to illustrate how the impacts may differ from those presented in the main text.

Table S3-8: Cases averted by one- and two-dose vaccination campaigns in Conakry with vaccination start days.

vaccination start day	mean (95% PI) cases averted with one-dose	mean (95% PI) ratio of cases averted 1-dose to 2-dose
40	3,975 (3,642-4,313)	1.10 (1.05-1.17)
55	3,393 (3,058-3,732)	1.16 (1.09-1.24)
70	1,865 (1,551-2,195)	1.25 (1.08-1.46)
85	749 (532-971)	†
100	217 (116-322)	†
115	64 (14-116)	†

†In some simulations there were no cases averted in the two-dose scenario so this ratio is not calculated.

Table S3-9 shows the results from simualtions where a single dose is 50% more and 50% less protective than in the main analysis. This highlights that a more protective single dose will shift the preferred strategy towards the use of one dose where as a less protective single dose does the opposite. However as seen in the other modeled settings, even with a vaccine with 22% efficacy, we find that there is no significant difference between the cases averted with one- and two-dose regimens.

Table S3-9: Results Cases Averted with Alternative RSEs in Conakry.

RSE (1-dose VE)	Mean Cases Averted (95% PI)	Ratio of Cases Averted (95% PI)
0.28 (0.22)	1,863 (1,489-2,243)	1.00 (0.84-1.16)
0.86 (0.83)	3,200 (2,888-3,535)	1.18 (1.11-1.26)

References

[1] Bretó C, He D, Ionides EL, King AA. Time Series Analysis via Mechanistic Models. The annals of applied statistics. 2009 Mar;3(1):319–348.

[2] Team RDC. R: A Language and Environment for Statistical Computing. Vienna, Austria; 2011.

[3] King AA, Ionides EL, Breto CM, Ellner SP, Ferrari MJ, Kendall BE, et al. pomp: Statistical inference for partially observed Markov processes (R package);.

[4] Reyburn R, Clemens JD, Deen JL, Grais RF, Bhattacharya SK, Sur D, et al. The Case for Reactive Mass Oral Cholera Vaccinations. PLoS Neglected Tropical Diseases. 2011 Jan;5(1):e952.

[5] Ionides EL, Bretó C, King AA. Inference for nonlinear dynamical systems. Proceedings of the National Academy of Sciences of the United States of America. 2006 Dec;103(49):18438–18443.

[6] Azman AS, Rudolph KE, Cummings DAT, Lessler J. The incubation period of cholera: a systematic review. The Journal of infection. 2013 May;66(5):432–438.

[7] Azman AS, Luquero FJ, Rodrigues A, Palma PP, Grais RF, Banga CN, et al. Urban cholera transmission hotspots and their implications for reactive vaccination: evidence from Bissau city, Guinea bissau. PLoS Neglected Tropical Diseases. 2012;6(11):e1901.

[8] Rinaldo A, Bertuzzo E, Mari L, Righetto L, Blokesch M, Gatto M, et al. Reassessment of the 2010-2011 Haiti cholera outbreak and rainfall-driven multiseason projections. Proceedings of the National Academy of Sciences of the United States of America. 2012 Apr;.

[9] Weil AA, Khan AI, Chowdhury F, LaRocque RC, Faruque ASG, Ryan ET, et al. Clinical Outcomes in Household Contacts of Patients with Cholera in Bangladesh. Clinical Infectious Diseases. 2009 Nov;49(10):1473–1479.

[10] Date KA, Vicari A, Hyde TB, Mintz E, Danovaro-Holliday MC, Henry A, et al. Considerations for oral cholera vaccine use during outbreak after earthquake in Haiti, 2010-2011. Emerging Infectious Diseases. 2011 Nov;17(11):2105–2112.
