# Supplementary material for: The Impact of a One-Dose versus Two-Dose Oral Cholera Vaccine Regimen in Outbreak Settings: A Modeling Study
Source: PLoS Med. 2015 Aug 25;12(8):e1001867. doi: 10.1371/journal.pmed.1001867 (PMC4549326; doi:10.1371/journal.pmed.1001867)
Supplement: S4 Text — (PDF) [file pmed.1001867.s005.pdf]

Performance of One- versus Two-Dose Oral Cholera Vaccine Campaigns in Response to Outbreaks: A Modeling Study
S4 Text: Sensitivity Analyses

Andrew S. Azman, Francisco J. Luquero, Iza Ciglenecki, Rebecca F. Grais, David A. Sack, and Justin Lessler

Contents

1 Impact of the Mixture of Fast and Slow Transmission Pathways **2**

2 Individual- versus Population-Level Protection for Alternative Vaccine Mechanisms **3**

3 Effect of the Reproductive Number on MRSE **5**

4 Impact of Delays in Vaccine-Derived Protection **6**

5 Impact of Inter-dose Timing **7**

1 Impact of the Mixture of Fast and Slow Transmission Pathways

Environmentally mediated transmission may play a significant role in some cholera epidemics.[1,2] Environmental transmission extends the time between subsequent generations of infection; significantly changing epidemic dynamics and potentially changing the impact of one and two-dose vaccination protocols.

To create comparable scenarios allowing us to explore the effect of transmission modes on the MRSE, we simulated epidemics where both pathways have the same reproductive number, thus:

$$\mathcal{R} = \frac{\beta}{\gamma} = \frac{n_{slow}\,\beta^{\star}}{\gamma^{\star}}$$

In our example epidemics (i.e., those calibrated to the 2009 epidemic in Bissau City, Guinea Bissau), increasing the proportion of slow transmission leads to an increase in the mean generation time and lengthens the time course of the epidemic, as shown in Figure S4-1. We explored the impact of the mix of fast and slow transmission on $MRSE$ and found that it has little effect on vaccination early on in an epidemic (green lines in Figure S4-2). However, when vaccinating later on in an epidemic, increasing the proportion of slow transmission increases $MRSE$ (orange lines in Figure S4-2).

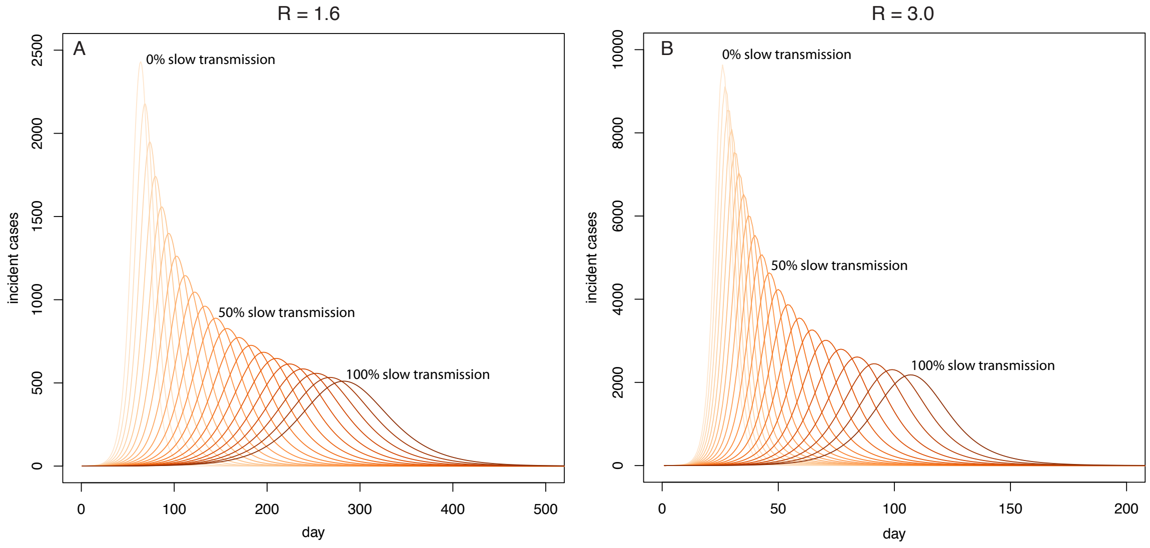

Figure S4-1: Uncontrolled epidemics with varying mixes of slow and fast transmission. Right and left panels illustrate epidemics with a reproductive number of 1.6 (A) and 3.0 (B), respectively.

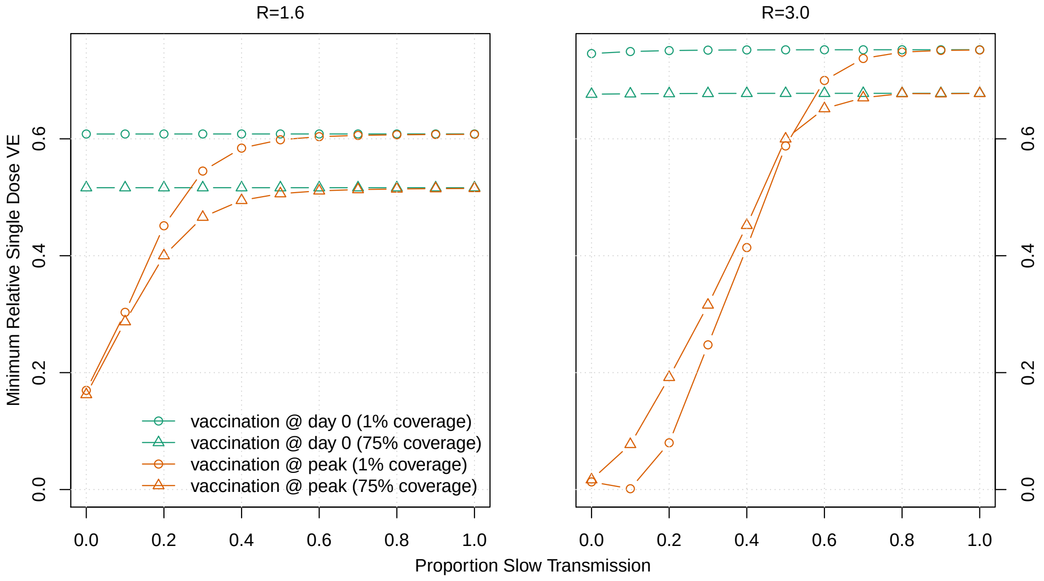

Figure S4-2: Impact of the mix of fast and slow transmission on the minimum single dose vaccine efficacy for epidemics with reproductive numbers of 1.6 (right) and 3.0 (left). Colors represent the timing of the vaccination campaign and the symbols represent the vaccine coverage (as defined by the proportion of the population that would be covered with a single dose).

We only explored this phenomenon within this deterministic framework and were unable to explore the impact on historic epidemics due to the lack of empirical data to support parameter assumptions and the inability to fit these parameters with the limited data available.

2 Individual- versus Population-Level Protection for Alternative Vaccine Mechanisms

In the main text we present the individual vs. population-level effects of one- vs. two-dose campaigns. Figures S4-3 and S4-4 illustrate similar plots for the severity-reducing and all-or-nothing vaccine models. Since the severity reducing vaccine simply protects against, disease and infectiousness, it does not change the risk of cholera infection for vaccinees which explains why the population and individual-level thresholds are identical.

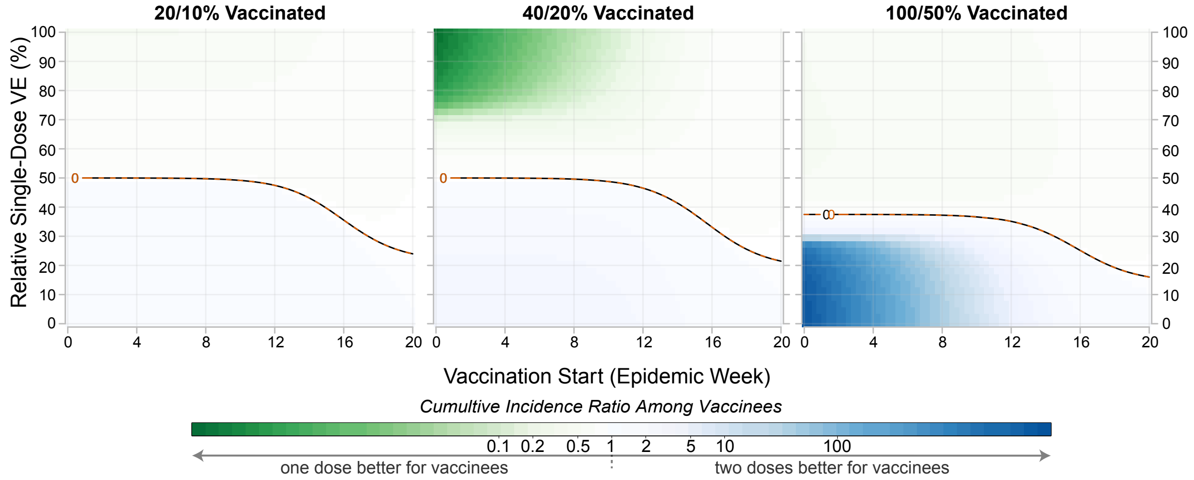

Figure S4-3: Comparison of individual and population-level benefits of one- and two-dose campaigns by vaccination start time and relative single-dose efficacy of a severity reducing vaccine. Colors in each panel represent the cumulative incidence ratio, comparing cumulative incidence among those ever receiving vaccine in one-dose campaigns (numerator) with the cumulative incidence among those ever receiving vaccine in two-dose campaigns (denominator). Black solid lines in each panel outline the region where single-dose campaigns are better for vaccinees (a result of indirect effects). Orange dashed lines represent the population-level threshold above which overall cumulative incidence is lower in a one-dose campaign compared to a two-dose campaign. Panels illustrate settings where enough severity reducing vaccine is available to cover 20%, 40%, and 100% of the population with a single dose.

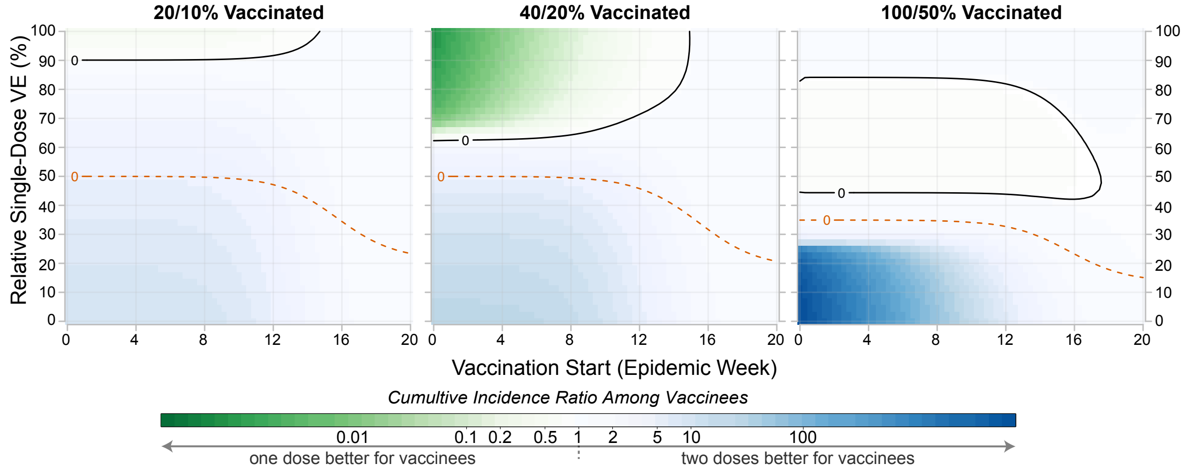

Figure S4-4: Comparison of individual and population-level benefits of one- and two-dose campaigns by vaccination start time and relative single-dose efficacy of an all-or-nothing vaccine. Colors in each panel represent the cumulative incidence ratio, comparing cumulative incidence among those ever receiving vaccine in one-dose campaigns (numerator) with the cumulative incidence among those ever receiving vaccine in two-dose campaigns (denominator). Black solid lines in each panel outline the region where single-dose campaigns are better for vaccinees (a result of indirect effects). Orange dashed lines represent the population-level threshold above which overall cumulative incidence is lower in a one-dose campaign compared to a two-dose campaign. Panels illustrate settings where enough severity reducing vaccine is available to cover 20%, 40%, and 100% of the population with a single dose.

3 Effect of the Reproductive Number on MRSE

In the main analysis we calibrated our model to data from an epidemic in Bissau City, Guinea Bissau,[3] where the reproductive number ($\mathcal{R}$) was 1.3. Figure S4-5 illustrates the impact of different reproductive numbers on the MRSE using different vaccination mechanisms with enough vaccine to cover the entire population with a single dose (i.e, 500,000 doses).

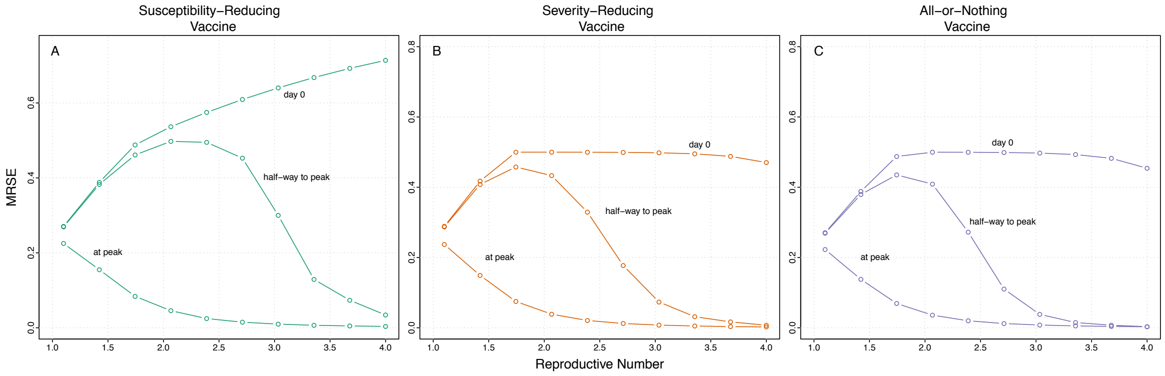

Figure S4-5: Each plot shows the $MRSE$ for varying basic reproductive numbers at three different times; the start of the epidemic (day 0), half-way to the epidemic peak of an uncontrolled epidemic ('half-way to peak'), and at the epidemic peak of an uncontrolled epidemic ('at peak'). Simulations were performed with 500,000 doses in a population of 500,000. Panels illustrate the impact of $\mathcal{R}$ on MRSE with different assumed vaccine mechanisms.

When vaccinating very early on (e.g., day 0 as shown in Figure S4-5), as $\mathcal{R}$ increases, each first dose provides less indirect protection thus raising the MRSE. At some threshold value of $\mathcal{R}$ (dependent on the vaccine mechanism, number of doses and vaccination day), the MRSE begins to decrease for higher values of $\mathcal{R}$ because the timing of the second dose shifts toward the end of the epidemic (since epidemics with higher $\mathcal{R}$'s are quicker) where few cases are averted. To help illustrate this effect, we show the epidemic curves (and cumulative incidence) in Figures S4-6 and S4-7 where we explore a range of reproductive numbers and MRSEs for a susceptibility reducing vaccine with vaccination starting on the first day of the epidemic.

As vaccination is delayed (e.g., the 'at peak' lines in Figure S4-5) , the dominant effect of increasing $\mathcal{R}$ consists of shifting the second dose closer to tail end of the epidemic where it eventually averts zero cases. Thus, increases in $\mathcal{R}$ later in the epidemic leads to a monotonically decreasing MRSE.

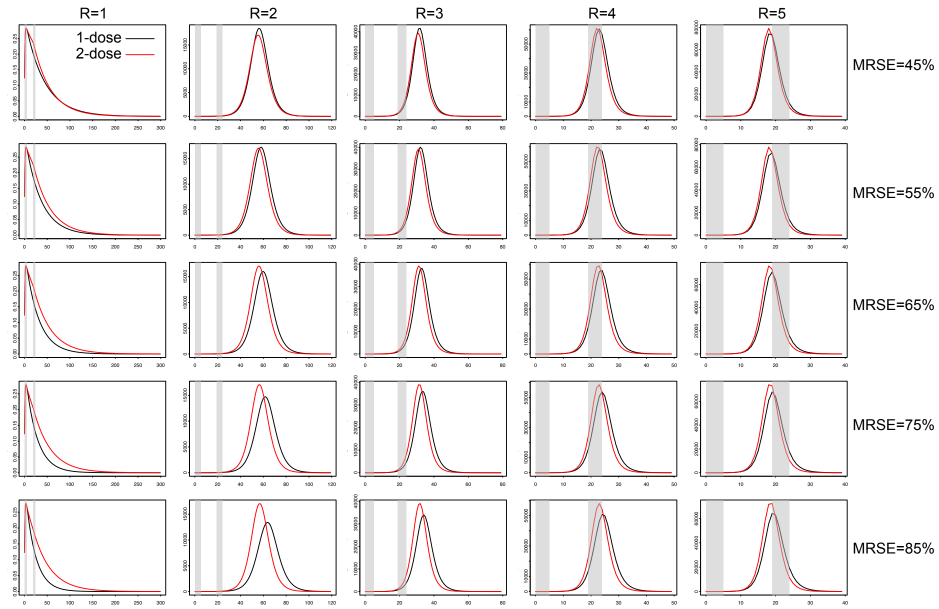

Figure S4-6: Illustration of the epidemic curves for simulated one- (black) and two-dose (red) epidemics for basic reproductive numbers ranging from 1 through 5 and MRSE values from 45-85%. Grey vertical strips indicate the vaccination periods in the two-dose campaign.

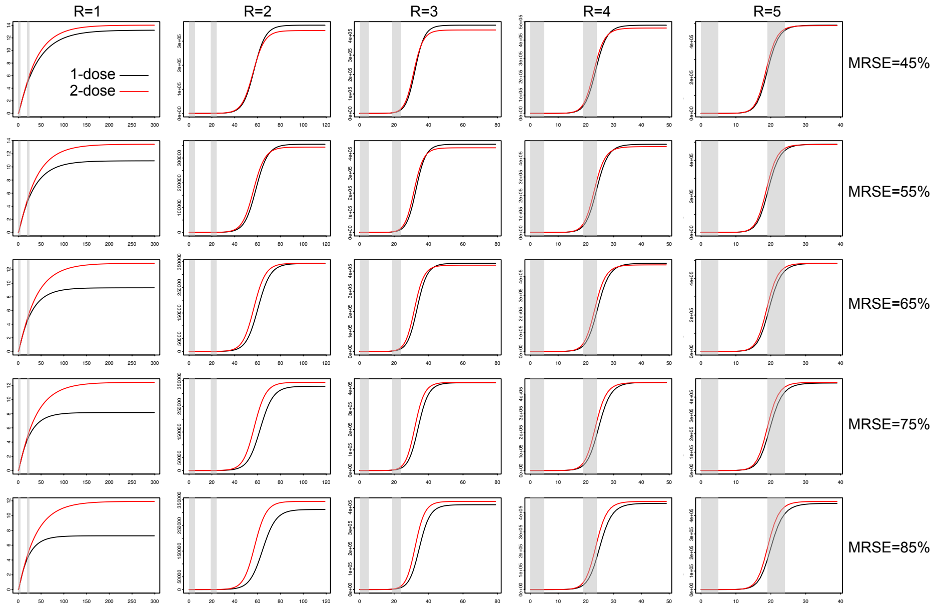

Figure S4-7: Illustration of the cumulative epidemic curves for simulated one- (black) and two-dose (red) epidemics for basic reproductive numbers ranging from 1 through 5 and MRSE values from 45-85%. Grey vertical strips indicate the vaccination periods in the two-dose campaign.

4 Impact of Delays in Vaccine-Derived Protection

In the main analysis onset of vaccine protection begins immediately after receiving the vaccine. In reality protection occur after some delay. In the simple case where the time to protection from each dose are assumed equal, our main results can simply be shifted by that number of days. For example, if the MRSE on day 20 of an epidemic was estimated to be 0.5 with protection starting immediately, and protection from each dose actually occurred

exactly 10 days after the vaccination, 0.5 would be the MRSE for day 10. The lag to protection, however, is likely to be differential after each dose with the lag after the first dose being the longest due to the development of the adaptive immune response. The actual time to protection for each dose is unknown so we explored the impact of the ratio of one-dose protection to two-dose protection on the MRSE. Figure S4-8 illustrates the effect that differential time to protection may have on the MRSE assuming a 14-day lag between receiving the first dose and onset of protection. In general, as the time to second-dose protection gets shorter compared to the time to first dose protection, the MRSE grows, thus favoring a two-dose protocol. Regardless of the delay, the maximum MRSE will be that of a proactive campaign (e.g. $MRSE = 0.5$ in this case, as shown by the lights color line in Panel B of Figure S4-8). In reality, these time lags will likely differ between settings, and may be influenced individuals previous exposure to cholera and general health status. While there are some data from hyper-endemic areas, like Bangladesh,[4] there are few little data from other areas to understand how long these delays actually are in places that see cholera less frequently.

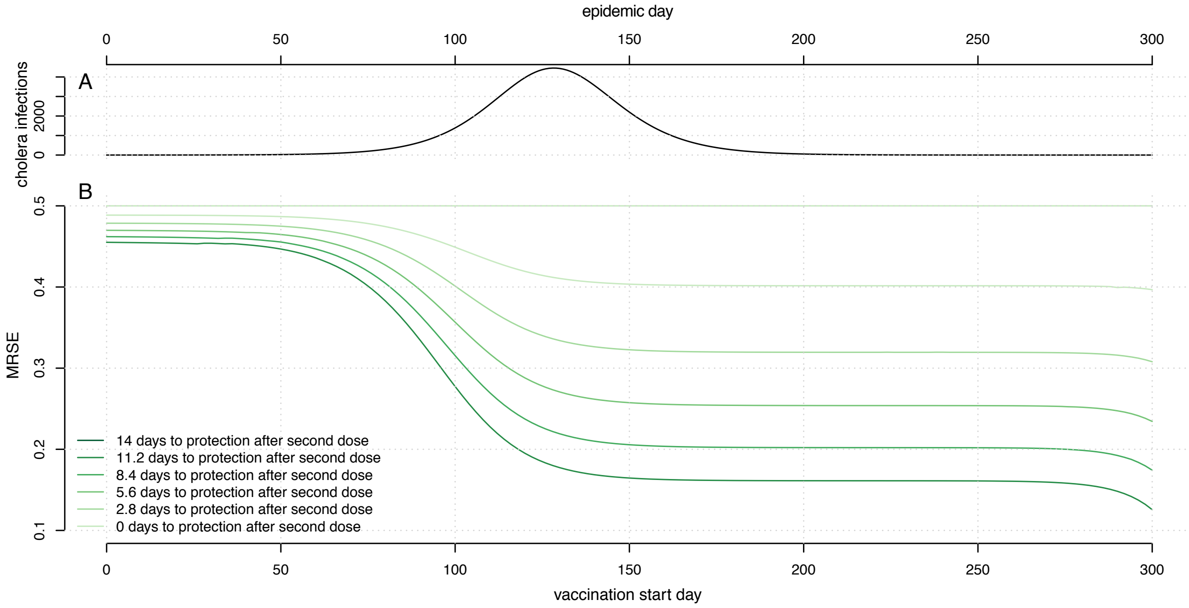

Figure S4-8: Impact of differential time to protection between first and second dose of OCV. Panel A shows the simulated epidemic curve. Panel B shows the MRSE as a function of vaccination start time (x-axis) for different lags between vaccination and onset of protection from the second dose, with darker green lines representing shorter lags. Each of these simulations assumed a 2-week lag between vaccination and protection for the first dose.

5 Impact of Inter-dose Timing

The timing between rounds was assumed to be the recommended 14-days in the main text. There are discussions about using different inter-dose timings so we explored the sensitivity of our results to changes in this this period from 7 to 28 days. Figure S4-9 illustrates how MRSE decreases more rapidly with vaccination delays when the inter-dose timing increases. For simplicity, these simulations assume protection from each dose is conferred immediately after vaccination.

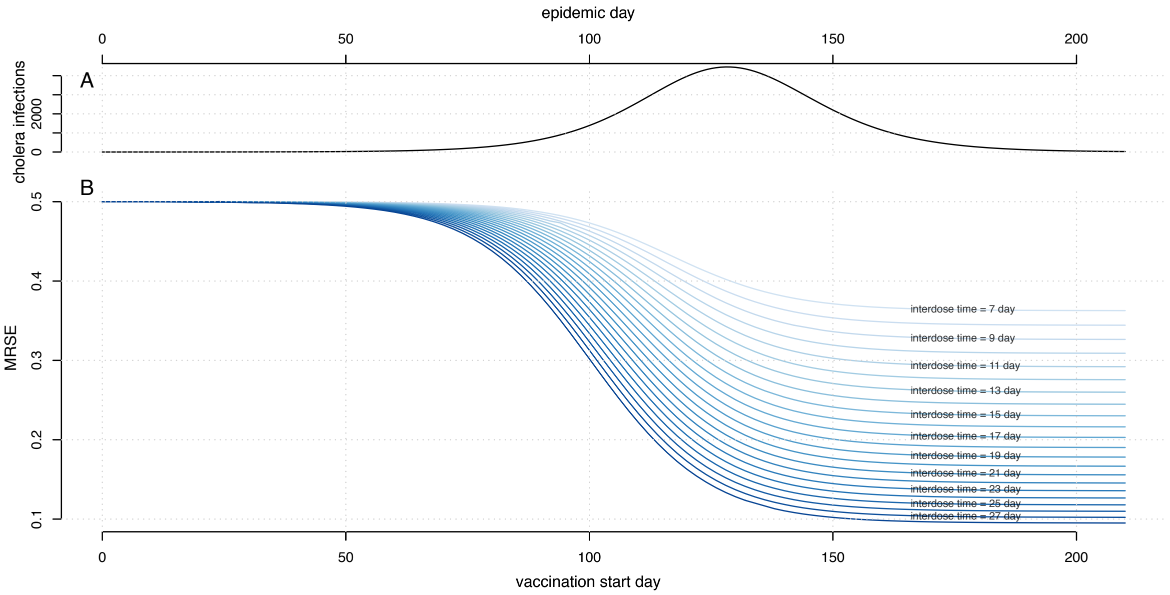

Figure S4-9: Impact of inter-dose timing on MRSE. Panel A shows the epidemic curve from unvaccinated simulations in black. Panel B illustrates the MRSE as vaccination is delayed with darker lines indicating a longer delay between doses.

References

[1] Bompangue D, Giraudoux P, Piarroux M, Mutombo G, Shamavu R, Sudre B, et al. Cholera Epidemics, War and Disasters around Goma and Lake Kivu: An Eight-Year Survey. PLoS Neglected Tropical Diseases. 2009 May;3(5):e436.

[2] Colwell RR, Huq A, Islam MS, Aziz KMA, Yunus M, Khan NH, et al. Reduction of cholera in Bangladeshi villages by simple filtration. Proceedings of the National Academy of Sciences of the United States of America. 2003 Feb;100(3):1051–1055.

[3] Luquero FJ, Banga CN, Remartínez D, Palma PP, Baron E, Grais RF. Cholera Epidemic in Guinea-Bissau (2008): The Importance of "Place". PloS one. 2011 May;6(5):e19005.

[4] Alam MM, Riyadh MA, Fatema K, Rahman MA, Akhtar N, Ahmed T, et al. Antigen-specific memory B-cell responses in Bangladeshi adults after one- or two-dose oral killed cholera vaccination and comparison with responses in patients with naturally acquired cholera. Clinical and vaccine immunology : CVI. 2011 May;18(5):844–850.
